# Machine Learning Identifies Metabolic Signatures that Predict the Risk of Recurrent Angina in Remitted Patients after Percutaneous Coronary Intervention: A Multicenter Prospective Cohort Study

**DOI:** 10.1002/advs.202003893

**Published:** 2021-03-08

**Authors:** Song Cui, Li Li, Yongjiang Zhang, Jianwei Lu, Xiuzhen Wang, Xiantao Song, Jinghua Liu, Kefeng Li

**Affiliations:** ^1^ Department of Cardiology Beijing Anzhen Hospital Capital University of Medical Sciences Beijing 100029 China; ^2^ Department of Cardiology Qufu People's Hospital Qufu Shandong 273100 China; ^3^ School of Medicine University of California San Diego CA 92093 USA

**Keywords:** machine learning, metabolomics, predictomes, recurrent angina, risk stratification

## Abstract

Recurrent angina (RA) after percutaneous coronary intervention (PCI) has few known risk factors, hampering the identification of high‐risk populations. In this multicenter study, plasma samples are collected from patients with stable angina after PCI, and these patients are followed‐up for 9 months for angina recurrence. Broad‐spectrum metabolomic profiling with LC‐MS/MS followed by multiple machine learning algorithms is conducted to identify the metabolic signatures associated with future risk of angina recurrence in two large cohorts (*n* = 750 for discovery set, and *n* = 775 for additional independent discovery cohort). The metabolic predictors are further validated in a third cohort from another center (*n* = 130) using a clinically‐sound quantitative approach. Compared to angina‐free patients, the remitted patients with future RA demonstrates a unique chemical endophenotype dominated by abnormalities in chemical communication across lipid membranes and mitochondrial function. A novel multi‐metabolite predictive model constructed from these latent signatures can stratify remitted patients at high‐risk for angina recurrence with over 89% accuracy, sensitivity, and specificity across three independent cohorts. Our findings revealed reproducible plasma metabolic signatures to predict patients with a latent future risk of RA during post‐PCI remission, allowing them to be treated in advance before an event.

## Graphical abstract

Plasma metabolomics followed by multiple machine learning algorithms and quantitative analysis is conducted to identify recurrent angina‐related alternations that reflect only a small subset of the plasma metabolome from cohort‐specific effects. This approach allows to develop and validate a robust and reproducible metabolic predictive model that can prospectively identify the future risk of angina recurrence across three independent cohorts.

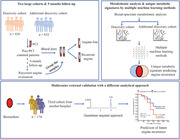

## 1 Introduction

Coronary artery disease (CAD) is the most common type of heart disease and the leading cause of mortality.^[^ ^1^ ^]^ Stable angina, a clinical symptom characterized by discomfort located in the chest, neck, or left arm, is a common manifestation of CAD.^[^ ^2^ ^]^ It was estimated that 15.8 million people in the United States have chronic CAD, and about half of them (> 8 million) have the symptom of stable angina.^[^ ^2^ ^]^ With the rapid development of interventional cardiology over the past decade, the number of myocardial revascularization procedures using coronary angioplasty in CAD patients has dramatically increased.^[^ ^3^ ^]^ Percutaneous coronary intervention (PCI) has now become the treatment of choice for CAD with stable angina.

Despite many improvements in coronary angioplasty techniques, angina recurrence after PCI is still a common problem that many cardiologists have to deal with in everyday clinical practice.^[^ ^4^ ^]^ About 20 to 30% of patients report the recurrence of angina after successful PCI with optimal medical therapy (OMT) at a one‐year follow‐up.^[^ ^5^ ^]^ The pathophysiological mechanisms of recurrent angina (RA) are complicated and have not been fully understood. Both structural and functional alterations after PCI might be involved.^[^ ^5^ ^]^ Several potential biomarkers, including circulating brain natriuretic peptide, plasma urokinase antigen, high‐sensitivity C‐reactive protein (hsCRP), and endothelial dysfunction, had been reported to have predictive values for angina recurrence.^[^ ^6^ ^]^ However, none of them were further validated using different cohorts. Strategies to enhance the prediction of Post‐PCI angina recurrence are urgently needed.

Recent advancements in omics and machine learning techniques have shown promising outcomes for individualized prediction and characterization of patients with CAD.^[^ ^7^ ^]^ Predictome is defined as the use of multivariate features from one or more omic modalities to predict the diseases.^[^ ^8^ ^]^ Among various omic techniques, metabolomics is a unique top‐down approach for studying complex systems as the metabolomic profile represents the phenotype of the cellular responses to physiological, pathophysiological stimuli, or epigenetic modification.^[^ ^9^ ^]^ Recently, metabolomics had been demonstrated to provide predictive information on acute myocardial infarction patients at high risk of death within two years after PCI,^[^ ^10^ ^]^ restenosis,^[^ ^11^ ^]^ and future CAD risk.^[^ ^12^ ^]^

In this study, we characterized the unique plasma metabolomic signatures associated with future angina recurrence in remitted patients after PCI in two large independent discovery cohorts. We developed a multi‐metabolite predictive model using ensemble machine learning methods from RA‐specific metabolic features to stratify the patients with the relapse of angina within nine months after PCI. The model was further validated using a cohort from another center with a different analytical approach. Our study will potentially help identify individuals at high‐risk who require more intensive therapy or, conversely, help avoid drug overuse and associated side effects in patients with a favorable prognosis.

## 2 Results

### 2.1 Patient Characteristics

**Figure 1:**
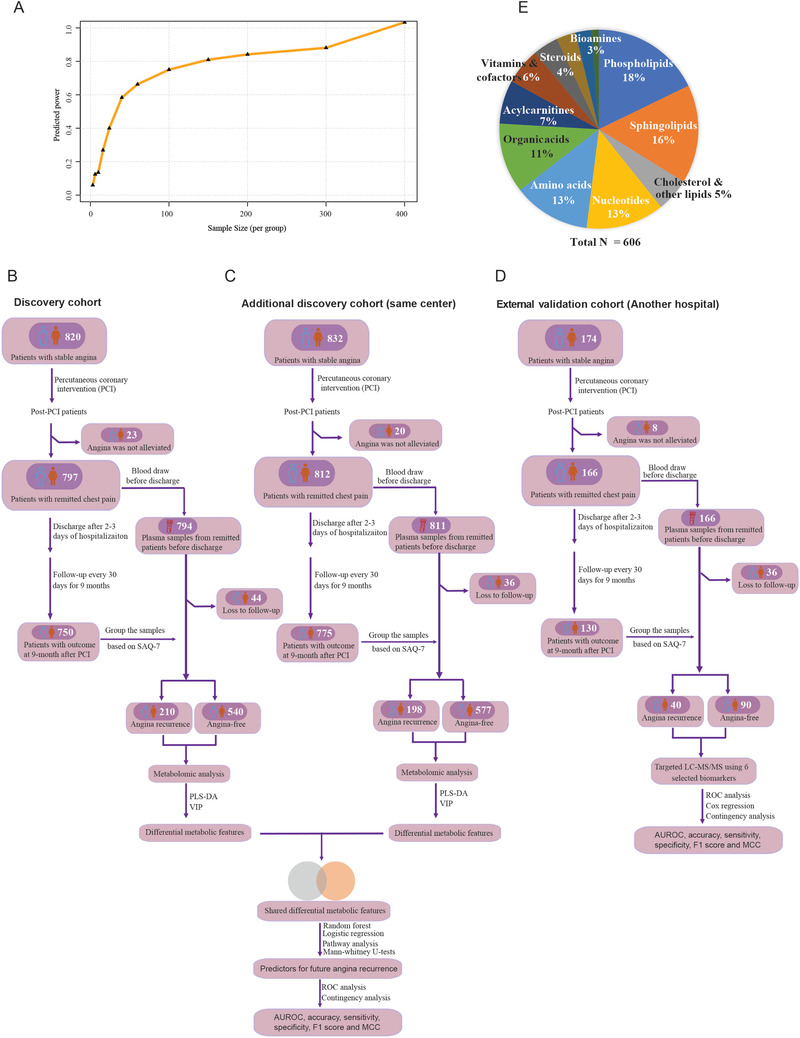
The experimental design for the study. A) Sample size estimation by power analysis to ensure the detection of true differences. The FDR was controlled at 0.05. B) The discovery cohort. Patients with stable angina were followed prospectively for nine months after PCI for the recurrence of angina. A total of 750 patients were used for metabolomic analysis. C) An additional independent discovery cohort from the same hospital (*n* = 775). D) The external validation cohort from another hospital (*n* = 130). E) A total of 606 metabolites covering the main chemical classes in human plasma were targeted.

We first performed a pilot study to calculate the minimum sample size to obtain the true statistical differences. The power analysis indicated that we have > 80% power to detect true discriminating features using about 140 subjects (**Figure** **1**A). Finally, three independent cohorts with a total of 1655 patients were included in this study. In the discovery cohort, of the 820 patients with stable angina, 797 patients had dramatic relief of angina with PCI and OMT. The plasma samples were collected before hospital discharge. The remitted patients were followed for angina recurrence, and 44 patients were lost to follow‐up. At nine months, 210 patients (28.0%) had a relapse of angina, and 540 were angina‐free (Figure 1B). The baseline characteristics were similar between patients with angina recurrence in remission and those with angina‐free (**Table** **1**). These baseline variables only had mild or moderate effect size (Hedges’ g < 0.5). Of the 832 patients assessed in the additional discovery cohort, angina was not alleviated in 20 cases with PCI and OMT, and 36 patients were lost to follow‐up. At nine months, 198 patients developed RA (25.5%), and 577 were angina‐free (Figure 1C and Table S1, Supporting Information). The external validation cohort was collected from another medical center, with a total of 130 patients enrolled in the analysis. At the end of follow‐up, 40 (30.8%) patients had angina recurrence, and 90 were angina‐free (Figure 1D and Table S2, Supporting Information).

**Table 1:** The characteristics of patients in the discovery cohort (*n* = 750)

Characteristics	Remitted patients with RA (*n* = 210)	Angina‐free (*n* = 540)	*P* value (RA vs Angina‐free)	Hedges′ g effect size (Angina vs Angina‐free)
Male (%)	100 (47.5%)	265 (49.1%)	0.66	0.029
Age (years)	59 ± 14.6	61 ± 13.8	0.08	0.14
BMI (kg m^−2^)	24.1 ± 6.1	23.3 ± 4.9	0.06	0.15
Smoking (n, %)	46 (22.2%)	101 (18.6%)	0.37	0.080
Hypertension	39 (18.5%)	82 (15.2%)	0.31	0.091
LDL‐C (mg dl^−1^)	96.8 ± 8.3	97.9 ± 12.1	0.23	0.10
HDL‐C (mg dl^−1^)	46.4 ± 8.5	47.3 ± 5.8	0.10	0.14
Uric acid (µmol L^−1^)	254 ± 94	265 ± 97	0.16	0.11
HbA1c (%)	5.6 ± 1.91	5.8 ± 1.53	0.14	0.12
hsCRP (mg dL^−1^)	0.13 (0.04–0.22)	0.15 (0.05–0.23)	0.15	0.34
Stent diameter (mm)	2.8 ± 0.63	2.9 ± 0.71	0.075	0.15
Ejection fraction (%)	61 ± 14.2	63 ± 15.9	0.11	0.023
Medications				
Aspirin	210 (100%)	540 (100%)	1.00	0.00
Clopidogrel	210 (100%)	540 (100%)	1.00	0.00
ACEI/ARB	36 (17.2%)	95 (17.6%)	0.81	0.012
*β*‐blocker	31 (14.8%)	83 (15.3%)	0.75	0.017
Statin	47 (22.2%)	113 (20.8%)	0.74	0.035

Notes: Values are mean ± SD, n (%), or median (interquartile range, IQR). Differences between angina and angina‐free groups were analyzed using Student's *t*‐test (parametric distribution), Mann–Whitney U test (nonparametric distribution), or two‐proportion *z*‐test (Categorical or proportional data). The effect size between the two groups was calculated by Hedge's statistic.

### 2.2 The Unique Metabolomic Features in the Plasma of Remitted Patients with Future Angina Recurrence

We performed metabolomic profiling of 606 metabolites covering the main chemical classes in human plasma for the discovery and internal validation cohorts (Figure 1E). The representative chromatogram for metabolomic profiling of a plasma sample is shown in Figure S2, Supporting Information. A total of 407 metabolites were detected in all the plasma samples with excellent analytical reproducibility as measured in replicate quality control (QC) samples throughout the analysis (median inter‐day coefficient variation [CV] of 11.5%) (Table S3, Supporting Information).

**Figure 2:**
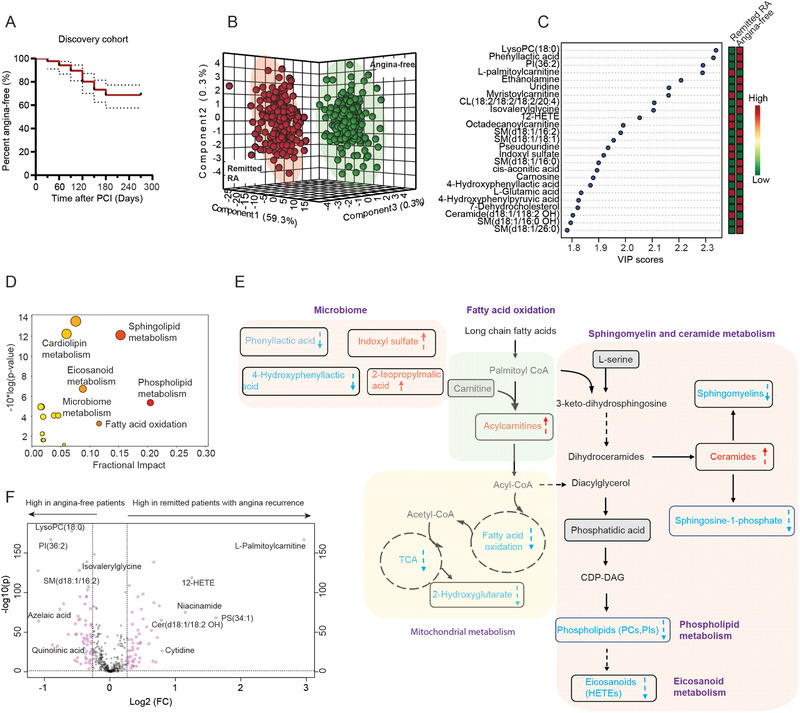
Metabolite and biochemical pathway abnormalities in remitted patients with future angina recurrence. A) Kaplan–Meier analysis of time to angina recurrence after PCI in the discovery cohort. Patients were evaluated for angina recurrence every 30 days for nine months after PCI. *n* = 210 for remitted RA and *n* = 540 for angina‐free patients. The dash lines are 95% CIs. B) PLS‐DA revealed the clear separation of the metabolomic profiles in the plasma of remitted patients with angina recurrence within nine months after PCI from those of angina‐free patients in the discovery cohort. C) The top 25 discriminating metabolites ranked by VIP scores in the discovery cohort. VIP scores ≥ 1.2 were considered statistically significant. D) Bubble impact plot of biochemical pathways disturbed in remitted RA compared with angina‐free patients for the discovery cohort. E) Detailed metabolic pathways altered in remitted patients with future angina recurrence in the discovery cohort. The red color and up arrow indicate an increase in metabolite levels. The blue color and down arrow indicate the decrease of metabolite levels. F) Volcano plot showing the univariate analysis of the significant differential metabolites between remitted patients with angina recurrence and angina‐free in the discovery cohort. Fold change (FC) and multiple *t*‐tests were performed. FC (Remitted RA/angina‐free) > 1.2. FDR adjusted *P* value < 0.05. *n* = 210 for Remitted RA and *n* = 540 for angina‐free. Abbreviations: PCI: Percutaneous coronary intervention; Remitted RA: Remitted patients with future recurrent angina; CI: confidence interval; PLS‐DA: Partial least square discriminant analysis; VIP: Variable importance in projection; FDR: False discovery rate.

The Kaplan–Meier analysis of time to angina recurrence after PCI in the discovery cohort is shown in **Figure** **2**A. The median time to recurrence after enrollment was 7 months (IQR: 5–8 months). Multivariate partial least squares discriminant analysis (PLS‐DA) revealed a distinct separation of the metabolomic profiles in the plasma of remitted patients with future RA from angina‐free patients in the discovery cohort (Figure 2B). Similarly, in the additional discovery cohort, remitted patients with future RA had a metabolic profile that could still be distinguished from angina‐free patients by multivariate analysis (Figure S3, Supporting Information). The reliability of the PLS‐DA model was validated using the leave‐one‐out cross‐validation (LOOCV) with Q^2^ values > 0.4 for both cohorts (Figures S4 and S5, Supporting Information). Top discriminating metabolites between angina and angina‐free for the discovery cohort are shown in Figure 2C. Despite fewer significantly different metabolites, metabolomic profiling revealed similar angina recurrence‐related alterations in the additional discovery cohort (Figure S6, Supporting Information). Pathway analysis revealed that alterations in lipid metabolism dominated the metabolic signature in remitted patients with future angina recurrence (Figure 2D). The top lipid pathways most affected were sphingolipids, phospholipids, fatty acid oxidation, and eicosanoids. The interconnection of key disturbed metabolic pathways is shown in Figure 2E. The findings obtained from the multivariate analysis were further confirmed by the univariate analysis, as shown in the volcano plot (Figure 2F).

### 2.3 Development of a Novel Multi‐Metabolite Model for Early Prediction of Future Angina Recurrence in Two Large Independent Discovery Cohorts

As it had previously been challenging to establish biomarker panels that could be replicated across cohorts, in this study, we performed broad‐spectrum metabolomic profiling for two large independent discovery cohorts to minimize the cohort‐specific effects. Metabolomic data are highly dimensional and often correlated. Machine learning can reduce the dimensionality of metabolomic data sets and improve the accuracy of risk prediction.^[^ ^13^ ^]^ We combined three machine learning methods, including PLS‐DA, random forest (RF), and Cox regression for feature reduction and selection. One of the advantages of this ensemble methodology is to obtain better predictive performance than could be obtained from any of the constituent learning algorithms alone.^[^ ^14^ ^]^

**Figure 3:**
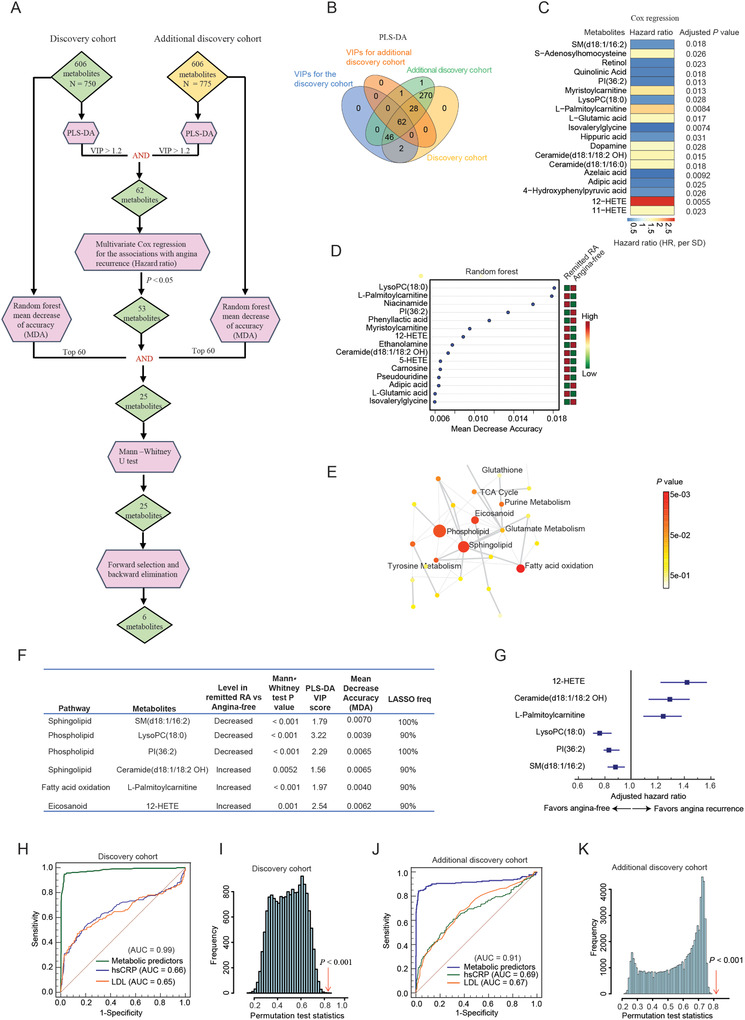
The development of a novel multi‐metabolite model for predicting future recurrence of angina using multiple machine learning algorithms in the discovery and additional independent replication cohorts. A) The flow chart of feature selection using the combination of multiple machine learning algorithms in two independent cohorts. “AND” indicates the intersection of two sets. B) Overlap of discriminating metabolites between the discovery (*n* = 750) and additional independent discovery cohorts (*n* = 775). The differential metabolites (Remitted RA vs angina‐free) in each cohort were defined as VIP scores ≥ 1.2 identified by PLS‐DA. C) Cox regression analysis revealed the top 20 metabolites that were significantly associated with future angina recurrence in the discovery cohort. HR are per 1 SD increment of log2‐transformed values. The multivariate analysis was adjusted for baseline age, BMI, hypertension status, hsCRPP, and LDL. D) The top 15 metabolites ranked by their contributions to classification accuracy (MDA scores) of angina recurrence in RF in the discovery cohort. *n* = 1000 trees. E) Enriched metabolite pathways associated with future angina recurrence. Each node represents a metabolite set with its color based on its *P* value. The hypergeometric test was performed to evaluate whether a particular metabolite set was represented more than expected by chance within the given library. Two‐tailed *P* < 0.05 and FDR ≤ 0.2 were considered as statistically significant. F) Six selected metabolic predictors and their biochemical pathways, *P* values for Mann–Whitney U tests, PLS‐DA VIP scores, MDA scores, and LASSO frequencies in the internal validation cohort. G) Associations of the selected metabolic predictors with future risk of angina recurrence in the additional discovery cohort. Multivariate Cox regression was performed, and the model was adjusted for baseline age, BMI, hypertension status, hsCRP, and LDL. HR are per 1 SD of log2‐transformed values. H) ROC curves generated by metabolic predictors, hsCRP, and LDL in the discovery cohort. I) The permutation test (*n* = 1000 times) was used to confirm the robustness of ROC analysis for the predictive model generated by metabolic signature in the discovery cohort. J) ROC curves generated by metabolic signature, hsCRP, and LDL for predicting future angina recurrence in the additional discovery cohort. K) The robustness of the predictive model generated by metabolic signature in the internal validation cohort was confirmed by the permutation test. Other characteristics are listed in Table 2. Abbreviations: Remitted RA: Remitted patients with recurrent angina. VIP: variance in projection; PLS‐DA: Partial least square discriminant analysis; SD: Standard deviation; MDA: Mean decrease accuracy; BMI: Body mass index; hsCRP: High‐sensitivity C‐reactive protein; LDL: Low‐density lipoprotein; ROC: Receiver operating characteristic; CI: Confidence interval. PI: Phosphatidylinositol; SM: Sphingomyelin; LysoPC: Lysophosphatidylcholine; MCC: Matthews correlation coefficient.

Because of the large number of features derived from metabolomic data, we first performed feature reduction using PLS‐DA for both discovery cohorts (**Figure** **3**A). Of 111 discriminating metabolites (angina vs angina‐free groups) _in the discovery cohort, 62 (55.9%) were replicated in the additional independent discovery cohort (Figure 3B). The full list of discriminating metabolites for the two cohorts are listed in Tables S4 and S5, Supporting Information. We next assessed the associations between the differential metabolites identified by PLS‐DA and future angina recurrence using multivariate Cox regression by controlling the baseline variables (Table S6, Supporting Information). Out of 62 shared differential metabolites in two cohorts, 53 were significantly associated with angina recurrence. The top 20 significant metabolic predictors associated with future angina recurrence are shown in Figure 3C, and the full list is in Table S7, Supporting Information. RF is another commonly used machine learning method for omics‐based biomarker discovery due to its resilience to high dimensionality data, insensitivity to noise, and resistance to overfitting.^[^ ^15^ ^]^ We then performed RF to identify which variables have more determinant impact to the prediction results in both the discovery and additional discovery cohorts. The importance of metabolites contributed to the classification between remitted patients with RA, and angina‐free is ranked by mean decrease accuracy (MDA) scores in Figure 3D and Figure S7, Supporting Information. Metabolites with top 60 MDA scores were selected and compared between two large discovery cohorts to find out the replicated metabolites (Tables S8 and S9, Supporting Information). Twenty‐five metabolites met the cut‐off criteria for all three machine learning methods in both cohorts (VIP ≥ 1.2 in PLS‐DA, *P* < 0.05 in Cox regression, and MDA scores of top 60).

Biomarker discovery requires not only the optimization of the biomarker usefulness regarding the biological relevance, but also the number of metabolites used in the predictive models. Given that metabolites in the same biochemical pathway might play similar roles in distinguishing RA patients from angina‐free subjects, we then performed enrichment analysis to identify the metabolic pathways of 25 metabolites identified by ensemble machine learning methods. As shown in Figure 3D, four biochemical pathways were enriched at *P* values < 0.05 and false discovery rates (FDRs) ≤ 0.2, including fatty acid oxidation (acylcarnitines), sphingolipid, phospholipid, and eicosanoid metabolism (Figure 3E). Using forward selection and backward elimination of metabolites from different pathways, we found that once a core set of six metabolites was selected, the addition of one or a few analytes had little effect on the overall quality of the classifier (Figure 3F). Among them, 12‐HETE, Ceramide(d18:1/18:2 OH), and L‐Palmitoylcarnitine were positively associated with increased risk of angina recurrence, while, LysoPC(18:0), PI(36:2), and SM(d18:1/16:2) had inverse associations with increased future RA risk in the additional discovery cohort (Figure 3G).

The predictive performance of the developed multi‐metabolite model for angina recurrence was evaluated by receiver‐operating characteristic curve (ROC) analysis. The routine clinical parameters hsCRP and low‐density lipoprotein (LDL) that were significantly associated with RA risk were also used for prediction analysis (Table S6, Supporting Information). Using the 6‐metabolite signature, we achieved an area under ROC (AUROC) of 0.99 (95% CI: 0.95–1.0), which is significantly higher than hsCRP (AUC of 0.66, 95% CI: 0.63–0.69), and LDL (AUROC of 0.65, 95% CI: 0.61–0.68) in the discovery cohort (Figure 3H). The predictive accuracy of metabolic biomarkers was further validated by the random permutation test with a *P* value < 0.001(Figure 3I). The accuracy, sensitivity, specificity, precision, F1 score, and Matthews correlation coefficient (MCC) in the discovery cohort were 97.6% (95% CI: 96.2–98.6%), 98.6% (95% CI: 95.9–99.7%), 97.2% (95% CI: 95.5–98.4%), 0.93, 0.96, and 0.94, respectively (**Table** **2**). Moreover, compared to the models constructed using the top 6 features selected from each single machine learning algorithm, the ensemble learning approach had a better predictive performance for future angina recurrence (Figure S8, Supporting Information).

**Table 2:** The performance of the developed multi‐metabolite model for predicting future angina recurrence after PCI

Stage	Accuracy [%] (95% CI)	Sensitivity [%] (95% CI)	Specificity [%] (95% CI)	Precision	F1 score	MCC
Discovery cohort	97.6 (96.2–98.6)	98.6 (95.9–99.7)	97.2 (95.5–98.4)	0.93	0.96	0.94
Additional discovery cohort	93.2 (91.2–94.9)	90.0 (85.1–93.7)	94.4 (92.2–96.2)	0.86	0.88	0.84
External validation from another hospital	89.2 (82.6–93.9)	87.5 (73.2–95.8)	90.1 (81.9–95.3)	0.80	0.83	0.76

The analyses were calculated by 2 × 2 contingency table analysis in R 4.0.3. PCI: Percutaneous coronary intervention; CI: Confidence interval; MCC: Matthews correlation coefficient.

Similarly, in the additional discovery cohort, the metabolic signature panel can reliably predict angina recurrence in advance with an AUC of 0.91 (95% CI: 0.87–0.94), superior to the traditional clinical parameters hsCRP and LDL (Figure 3J,K). The accuracy, specificity, precision, F1 score, and MCC were consistently over 90% or higher than 0.8 (Table 2).

### 2.4 External Validation of the Developed Multi‐Metabolite Model for Predicting Angina Recurrence

**Figure 4:**
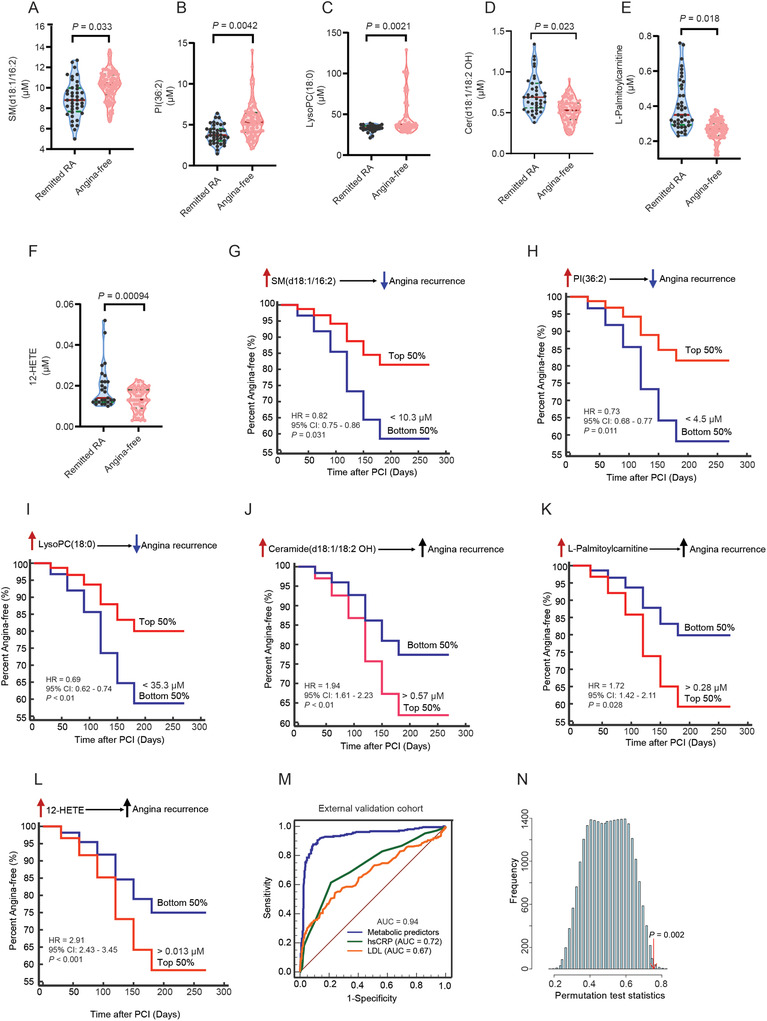
The external validation of the developed multi‐metabolite model for predicting future angina recurrence in remitted patients after PCI using a cohort from another hospital and a different analytical platform. A–F) Quantitative analysis of six metabolic predictors using a stable‐isotope dilution LC‐MS/MS method. *n* = 40 for remitted RA and *n* = 90 for angina‐free. In each violin plot, the red line represents the median value, and the green lines are two quartile lines (75% and 25%). Mann–Whitney U test was performed to compare the group differences. G–L) Cox regression analysis of the associations between metabolic predictors and future angina recurrence within nine months after PCI. HR are per 1SD of log2‐transformed values. The model was adjusted for baseline age, BMI, hypertension status, hsCRP, and LDL. M) ROC curve analysis to evaluate the performance of the predictive models generated by metabolic predictors, hsCRP, and LDL in the external validation cohort. N) The permutation test (*n* = 1000 times) was used to confirm the robustness of ROC analysis for metabolic predictors. Other characteristics are listed in Table 2. Abbreviations: PCI: Percutaneous coronary intervention; LC‐MS/MS: Liquid chromatography coupled with tandem mass spectrometry; Remitted RA: Remitted patients with recurrent angina; HR: Hazard ratio; BMI: Body mass index; hsCRP: High‐sensitivity C‐reactive protein; LDL: Low‐density lipoprotein; ROC: Receiver operating characteristic; MCC: Matthews correlation coefficient.

We next performed another independent validation of the developed model using a cohort from a different medical center. Similar to the other two cohorts, the median time to angina recurrence after enrollment in the external validation cohort was seven months (IQR: 5–8 months) (**Figure** **4**A). Quantitative analysis of biological fluids by LC‐MS/MS is the gold standard technique for preventive and diagnostic care.^[^ ^16^ ^]^ To verify the findings of metabolomic profiling in the discovery and additional discovery cohorts, we developed a new stable‐isotope dilution LC‐MS/MS method to quantify the selected metabolic predictors in the plasma of the external validation cohort, which is more quantitatively sound with greater clinical utility than metabolomic assessments. The details for method development and validation are described in Table S10, Figures S9 and S10, Supporting Information.

Quantitative alternations of the metabolic signature panel were consistent across the cohorts with significantly lower levels of LysoPC(18:0), PI(36:2), SM(d18:1/16:2), and higher levels of 12‐HETE, Ceramide(d18:1/18:2 OH), and L‐Palmitoylcarnitine in the plasma of remitted patients with future RA than those in patients with angina‐free (All *P* values < 0.05) (Figure 4B–F). When the subjects were stratified into top and bottom 50th percentiles, the remitted patients in the top 50th percentile for SM(d18:1/16:2) were much slower to experience recurrence (8 ± 1 vs 4 ± 1 months, HR = 0.82, 95% CI: 0.75–0.86, *P* = 0.031) (Figure 4G). Increased level of PI(36:2) (HR = 0.73, 95% CI: 0.66–0.77, *P* = 0.011) and LysoPC(18:0) (HR = 0.69, 95% CI: 0.62–0.74, *P* < 0.01) decreased recurrence risk (Figure 4H,I). In contrast, patients with higher level of Ceramide(d18:1/18:2 OH)(>0.57 µM, HR: 1.94, 95% CI: 1.61–2.23, *P* < 0.01), L‐Palmitoylcarnitine (>0.28 µM, HR = 1.72, 95% CI: 1.42–2.11, *P* = 0.028), and 12‐HETE (> 0.013 µM, HR = 2.91, 95% CI: 2.43–3.45, *P* < 0.001) were more likely to develop angina recurrence within nine months after PCI (Figure 4J–L).

Finally, we used multivariate logistic regression to build a multi‐metabolite model in the external validation cohort. The coefficients of 6 selected biomarkers were −2.269 (12‐HETE), −1.536 (L‐Palmitoylcarnitine), −0.961 [Ceramide (d18:1/18:2 OH)], 1.508 [LysoPC (18:0)], 0.836 [SM(d18:1/16:2)], and 0.787 [(PI 36:2)] (Table S11, Supporting Information). In addition, ROC analysis showed that the 6‐metabolite predictive model performed well in the external validation cohort with an AUC of 0.94 (95% CI: 0.82–0.99), which is significantly higher than hsCRP of 0.72 (95% CI: 0.69–0.76) and LDL of 0.67 (95% CI: 0.63–0.71) (Figure 4M,N). The accuracy, sensitivity, specificity, precision, F1 score, and MCC were 89.2% (95% CI: 82.6–93.9%), 87.5% (95% CI: 73.2–95.8%), 90.1% (95% CI: 81.9–95.3%), 0.8, 0.83, and 0.76, respectively in the external validation cohort (Table 2).

## 3 Discussion

Our metabolomic analysis revealed an underlying metabolic signature in the plasma of remitted patients with future angina recurrence that distinguished them from angina‐free controls. This difference was clinically inapparent as the plasma samples were collected during remission. Patterns of metabolic abnormalities that we found reflected alterations in chemical communication across lipid membranes and the nexus of mitochondria. Lipid abnormalities constituted the largest proportion of the metabolic signature associated with RA. Even more striking was the finding that metabolomic analysis was able to unmask a latent signature of future risk of angina recurrence with over 89% accuracy in three prospective cohorts. Our study could have a significant impact on clinical practice by permitting patients with stable angina to be stratified according to future risk of relapse after coronary revascularization and new preventive treatments to be tested. To our knowledge, this study is the first to identify a metabolic signature that predicts future angina recurrence.

A general challenge in biomarker discovery studies using the conventional “triangular” approach is cohort‐specific effects, which are particularly related to the multicenter studies. In this study, we used a modified “rectangular” strategy,^[^ ^17^ ^]^ by which broad‐spectrum metabolomic profiling was conducted on two large independent cohorts. Both cohorts were then analyzed together by machine learning algorithms. Machine learning allowed us to reduce the dimensionality of data sets and improve the accuracy of models for risk prediction.^[^ ^18^ ^]^ We used an ensemble‐based approach by the combination of multiple machine learning methods for feature reduction and selection. Ensemble‐based approaches are based on the idea that group decisions can take advantage of the strengths of individual algorithms, overcome their weaknesses, and achieve more robust results.^[^ ^14^ ^]^ The developed multi‐metabolite model was further validated using a stable‐isotope diluted LC‐MS/MS method in the third cohort from another center. We note that such targeted platforms are more quantitatively sound with greater clinical utility than metabolomic assessments. Collectively, our strategy allows discerning RA‐related alternations that reflect a small subset of the plasma metabolome from other cohort‐specific effects comprising larger parts of the quantified plasma metabolome.

The prognostic metabolites that were found to regulate the risk of angina recurrence were from several lipid pathways, including eicosanoid (HETEs), sphingolipid, phospholipid, and acylcarnitines. High 12‐HETE was the most predictive risk factor for future angina recurrence in remitted patients. HETEs are a family of oxylipins formed from 20‐carbon polyunsaturated fatty acids such as arachidonic acid (C20:4) by non‐enzymatic lipid peroxidation or lipoxygenases (LOXs) under stress.^[^ ^19^ ^]^ These arachidonic acid‐derived HETEs are involved in various inflammatory pathologies acting as lipid mediators.^[^ ^20^ ^]^ Higher levels of pro‐inflammatory mono‐HETEs have been previously associated with the risks of coronary heart disease^[^ ^21^ ^]^ and atherosclerosis,^[^ ^22^ ^]^ but not RA. These findings support the idea that early mechanisms involving lipid oxidation, for which these metabolites may be part of the cause or a consequence, may contribute to the etiology of RA before an event.

The importance of sphingolipids (sphingomyelins and ceramides) in cardiovascular diseases has been underscored by several recent studies.^[^ ^23^ ^]^ Besides the significant associations with angina recurrence discovered in our study, elevated ceramides were reported as biomarkers of CAD,^[^ ^24^ ^]^ risk predictors for incident CVD,^[^ ^25^ ^]^ heart failure,^[^ ^26^ ^]^ atrial fibrillation,^[^ ^27^ ^]^ and mortality of CVD.^[^ ^28^ ^]^ Additionally, these metabolites have both structural roles as the building blocks of the plasma membrane and signaling functions for chemical communications across lipid membranes between cells. During growth conditions, ceramide is converted to sphingomyelin in the Golgi by sphingomyelin synthase, which is then transported in distinct vesicles to the plasma membrane.^[^ ^29^ ^]^ In contrast, stress stimuli promote the breakdown of sphingomyelin by sphingomyelinases, and the produced ceramide is involved in multiple biological processes that may influence angina recurrence, including inflammation,^[^ ^23^ ^]^ endothelial dysfunction,^[^ ^30^ ^]^ and apoptosis.^[^ ^31^ ^]^ Furthermore, our study suggested that 2’‐hydroxy (2’‐OH) ceramides could be more robust predictors than ceramides themselves for angina recurrence. The 2’‐hydroxylation of the fatty acid precursor of the amide acyl chain of sphingolipids is catalyzed by the peroxisomal enzyme fatty acid 2‐hydroxylase (FA2H).^[^ ^32^ ^]^ 2’‐OH ceramides had been largely ignored in cardiovascular diseases, and further studies are warranted to investigate the biological mechanisms of ceramide hydroxylation in RA.

Acylcarnitines are fatty acid derivative esters of carnitine, which mediate the import of long‐chain and medium‐chain fatty acid from the cytoplasm into mitochondria for beta‐oxidation. These metabolites are particularly abundant in the myocardium and skeletal muscle, which preferentially use fatty acid to generate energy. Acylcarnitines may leave the mitochondria through carnitine‐acylcarnitine translocase and enter the bloodstream. When mitochondrial fatty acid oxidation is impaired, several acylcarnitines are accumulated and elevated in the blood. The circulating acylcarnitine levels were shown to be correlated with those in heart tissues,^[^ ^33^ ^]^ and increased long‐chain acylcarnitines in plasma and serum had potential prognostic values for cardiovascular death^[^ ^34^ ^]^ and heart failure.^[^ ^35^ ^]^ In this study, plasma acyl‐carnitines were increased in remitted patients with RA. Our observation indicated that even in remitted patients, mitochondrial dysfunction may persist and play roles in angina recurrence.

Our study provided a novel model for the risk stratification of patients with RA in remission. If eicosanoids, phospholipids, sphingolipids, and acylcarnitines are addressed together, more than 80% of the metabolic risk of recurrence might be amenable to intervention. Recent studies have demonstrated that some antidepressants such as fluoxetine can inhibit the stress‐related conversion of sphingomyelin to ceramide,^[^ ^36^ ^]^ decrease HETEs,^[^ ^37^ ^]^ and restores the normal acylcarnitine levels.^[^ ^38^ ^]^ Additionally, previous meta‐analyses showed that treatment with antidepressants might improve CVD prognosis.^[^ ^39^ ^]^ New clinical trials designed to test the potential benefits of certain antidepressants in reducing the risks of angina recurrence after PCI could be the next step in applying the results of this study.

Our study has several major strengths. First, this is a large, multicenter, prospective study with extensive clinical information and low rates of dropout. Second, we conducted a “rectangular” strategy followed by the integration of several advanced machine learning algorithms for generating a robust and reproducible predictive model. Third, the selected metabolic predictors are from several different biochemical pathways, which may provide a more accurate and comprehensive readout of disease status. Our approach, therefore, limits the identification of spurious or cohort‐specific associations.

Some limitations warrant discussion. The underlying causes of RA are sophisticated. Although we had carefully adjusted for potential covariates, residual confounding cannot be ruled out. Moreover, the age and ethnic homogeneity of our sample population might limit the generalizability of our findings to other populations. Additional validation in a more diverse age and demographic group is necessary to confirm our results.

## 4 Conclusion

In summary, based on the reproducible findings in three independent cohorts, the present study identified RA‐specific abnormalities in remitted patients after PCI dominated by alternations in lipid biochemical pathways, including sphingolipid, phospholipid, eicosanoid, and fatty acid oxidation. We developed and validated a novel multi‐metabolite predictive model from this unique metabolic signature that can prospectively identify the recurrence risk of angina with over 89% accuracy. The inclusion of these risk biomarkers in a clinician's armamentarium has the potential to greatly improve the ability to identify at‐risk patients and enable early intervention and potentially a reduction of post‐PCI angina recurrence.

## 5 Experimental Section

##### Study Population

This multicenter prospective cohort study was approved by the Institutional Review Boards (IRBs) of Beijing Anzhen Hospital (IRB#: AZHEC2012‐0516) and Qufu People's Hospital (IRB#: 224718‐3) and conformed to the World Medical Association Declaration of Helsinki‐Ethical Principles for Medical Research Involving Human Subjects. The study design and data analysis were performed according to the Standards for Reporting Recommendations for Tumor Marker Prognostic Studies.^[^ ^40^ ^]^ The inclusion criteria were hospitalized CAD patients with stable angina. Each patient received one second‐generation rapamycin‐eluting stent. In addition to PCI, all patients were given OMT. The remitted patients after treatment were recruited (Figure S1, Supporting Information). More detailed inclusion and exclusion criteria can be found in the Supporting Information.

Total 750 patients in Beijing Anzhen Hospital were enrolled between December 2015 and May 2016 as the discovery cohort (cohort 1) and another 775 patients from February 2017 to August 2017 for the additional independent discovery cohort (cohort 2). The external validation cohort (cohort 3) was recruited in Qufu Hospital, Shandong Province, from January 2018 through June 2018. The written informed consent was obtained from all subjects.

##### Blood Collection and Patent's Follow‐Up for Angina Recurrence

After an overnight fast, venous blood was collected from each patient at 48 h after PCI. The patients were discharged after blood draw and followed up every 30 days for angina recurrence for nine months. The Chinese version of the Seattle angina questionnaire‐7 was used to evaluate the severity of angina.^[^ ^41^ ^]^ The plasma samples were then grouped into RA and angina‐free based on the status of angina recurrence at the end of the follow‐up.

##### Metabolites Extraction and Metabolomic Analysis for the Discovery and Internal Validation Cohorts

Briefly, 95 µL of plasma was thawed, mixed with 5 µL of custom‐synthesized stable isotope standards, and extracted with 400 µL of prechilled (−20 °C) extraction buffer containing acetonitrile and methanol (50: 50, v/v).^[^ ^42^ ^]^ The supernatant after protein precipitation and centrifugation was used for further analysis. Metabolomic analysis was performed on a Shimadzu LC‐20A ultra‐high‐performance liquid chromatography coupled with SCIEX QTRAP 6500+ triple quadrupole mass spectrometer (LC‐MS/MS) using a broad‐spectrum targeted metabolomic approach.^[^ ^43^ ^]^ In total, 606 metabolites covering the major chemical classes in human plasma were targeted by scheduled multiple reaction monitoring. The pooled plasma samples were used as QC throughout the analysis. Method details are provided in the Supporting Information.

##### External Validation Using the Patients from a Different Medical Center with a Different Analytical Platform

To further validate the developed multi‐metabolite prediction model, absolute quantification of the metabolic predictors was performed in the external validation cohort by stable‐isotope dilution LC‐MS/MS. The concentration of the targeted metabolites was calculated from the calibration curves. Method details are provided in the Supporting Information.

##### Statistical Analysis

Subject characteristics: data were reported as mean ± standard deviation (SD) or as the median and interquartile range (IQR) (Skewed data). Differences between RA and angina‐free groups were analyzed using either Student's *t*‐tests or Mann–Whitney *U* tests. The proportions between the two groups were analyzed using the two‐proportion *z*‐test. The effect size was calculated using the Hedge's statistic. *P* < 0.05 was statistically significant. Time to angina recurrence was analyzed by the Kaplan–Meier analysis in MedCalc 19.1.3.

Metabolomic data analysis: metabolomic data were log_2_ transformed and scaled by control SDs for further analysis. Power analysis was conducted to estimate the number of samples (sample size) necessary to identify statistically significant metabolites between angina and angina‐free groups from a small pilot cohort according to a previously described method.^[^ ^44^ ^]^ The sample size for each individual metabolite was calculated using the following equation:

(1) $$\frac{\left(r+1\right)\sigma^{2}\times {\left(Z_{\frac{\alpha }{2}}+Z_{\beta }\right)}^{2}}{r{\left(\mu 1-\mu 2\right)}^{2}}$$

*r* is the ratio of the larger group to the smaller group. *σ* is the SD of the peak area for a metabolite in the population. *Z*_*α*/2_ is the standard normal *z*‐value for a significance level *α* = 0.05, which is 1.96. Z_*β*_ is the standard normal *z*‐value for the power of 80%, which is 0.84. µ1−µ2 is the difference in means of the metabolite peak area between the two groups. The average power of all metabolites was then calculated while controlling the FDR at 0.05.

Multivariate PLS‐DA was performed in MetaboAnalyst 5.0. The validation of the PLS‐DA model was performed via LOOCV. Metabolites with PLS‐DA variable importance in projection (VIP) scores ≥ 1.2 were statistically significant. Metabolic pathway and enrichment analyses were performed using a custom Python script with an in‐house library for human metabolism. The hypergeometric test was used to evaluate whether a particular metabolite set was represented more than expected by chance within the given library. Two‐tailed *P* values were calculated after adjusting for multiple testing. *P* < 0.05 and FDR ≤ 0.2 were considered as statistically significant.

Feature selection, predictive model construction, and validation: Three machine learning methods were used for the selection of optimal features in R 4.0.3 with MetaboAnalystR 3.0 package.^[^ ^45^ ^]^ The shared significant metabolites identified by PLS‐DA in both the discovery and internal validation cohorts were used for further analysis. A decision tree‐based ensemble method (RF) was also performed with 1000 trees. Additionally, multivariate Cox regression was used to assess the association of log_2_‐transformed metabolite intensities with angina recurrence, adjusting for age, body mass index (BMI), the presence of hypertension, hsCRP, and LDL. Hazard ratios (HR) were per 1 SD increment of log‐transformed values. The least absolute shrinkage and selection operator (LASSO) was conducted for auto feature selection, and the selection frequencies for significant metabolites were obtained. A forward selection and backward elimination method was then used to determine the optimal set of features.^[^ ^46^ ^]^ A multi‐metabolite model was built by multivariate logistic regression analysis adjusted for age, BMI, the presence of hypertension, hsCRP, and LDL.

The performance of the predictive model was assessed using the ROC analysis, and 95% confidence intervals (CIs) for the area under ROC curves (AUROC) were calculated by bootstrap resampling (100 times). Classifier robustness was estimated by permutation tests (1000 times). Sensitivity, specificity, and accuracy were calculated by 2 × 2 contingency table analysis. The F1 score and MCC were calculated in R 4.0.3, which provided complementary information to AUROC, especially with class‐imbalanced datasets. All general statistical analyses were performed in GraphPad Prism 9.0 (San Diego, CA), MedCalc 19.1.3. (Ostend, Belgium), and R 4.0.3 unless specified in the methods and legends. A *P* value of two sides less than 0.05 was considered statistically significant.

## Conflict of Interest

The authors declare no conflict of interest.

## Data Availability

The raw metabolomic data generated from this study has been uploaded to NIH Metabolomics Workbench (Accession ID: ST001420).

## Supporting information

Supporting Information

## References

[1] E. J. Benjamin , M. J. Blaha , S. E. Chiuve , M. Cushman , S. R. Das , R. Deo , S. D. de Ferranti , J. Floyd , M. Fornage , C. Gillespie , C. R. Isasi , M. C. Jimenez , L. C. Jordan , S. E. Judd , D. Lackland , J. H. Lichtman , L. Lisabeth , S. Liu , C. T. Longenecker , R. H. Mackey , K. Matsushita , D. Mozaffarian , M. E. Mussolino , K. Nasir , R. W. Neumar , L. Palaniappan , D. K. Pandey , R. R. Thiagarajan , M. J. Reeves , M. Ritchey , C. J. Rodriguez , G. A. Roth , W. D. Rosamond , C. Sasson , A. Towfighi , C. W. Tsao , M. B. Turner , S. S. Virani , J. H. Voeks , J. Z. Willey , J. T. Wilkins , J. H. Wu , H. M. Alger , S. S. Wong , P. Muntner , American Heart Association Statistics Committee and Stroke Statistics Subcommittee, Circulation 2017, 135, e146. 28122885 10.1161/CIR.0000000000000485 PMC5408160

[2] T. C. Trujillo , P. P. Dobesh , Pharmacotherapy 2007, 27, 1677. 18041888 10.1592/phco.27.12.1677

[3] D. T. Ko , J. V. Tu , Z. Samadashvili , H. Guo , D. A. Alter , W. J. Cantor , E. L. Hannan , Circulation 2010, 121, 2635. 20529997 10.1161/CIRCULATIONAHA.109.926881

[4] P. Izzo , A. Macchi , L. De Gennaro , A. Gaglione , M. Di Biase , N. D. Brunetti , Eur. Heart J. Acute Cardiovasc. Care 2012, 1, 158. 24062904 10.1177/2048872612449111 PMC3760523

[5] G. Niccoli , R. A. Montone , G. A. Lanza , F. Crea , Int. J. Cardiol. 2017, 248, 14. 28807510 10.1016/j.ijcard.2017.07.105

[6] H. Takase , T. Toriyama , T. Sugiura , R. Ueda , Y. Dohi , Eur. J. Clin. Invest. 2004, 34, 79. 14764069 10.1111/j.1365-2362.2004.01301.x

[7] Y. Fan , Y. Li , Y. Chen , Y. J. Zhao , L. W. Liu , J. Li , S. L. Wang , R. N. Alolga , Y. Yin , X. M. Wang , D. S. Zhao , J. H. Shen , F. Q. Meng , X. Zhou , H. Xu , G. P. He , M. D. Lai , P. Li , W. Zhu , L. W. Qi , J. Am. Coll. Cardiol. 2016, 68, 1281. 27634119 10.1016/j.jacc.2016.06.044

[8] B. Rashid , V. Calhoun , Hum. Brain Mapp. 2020, 41, 3468. 32374075 10.1002/hbm.25013 PMC7375108

[9] J. Wang , Y. Sun , S. Teng , K. Li , BMC Med. 2020, 18, 83. 32290837 10.1186/s12916-020-01546-5 PMC7157979

[10] A. Vignoli , L. Tenori , B. Giusti , P. G. Takis , S. Valente , N. Carrabba , D. Balzi , A. Barchielli , N. Marchionni , G. F. Gensini , R. Marcucci , C. Luchinat , A. M. Gori , BMC Med. 2019, 17, 3. 30616610 10.1186/s12916-018-1240-2 PMC6323789

[11] S. Cui , K. Li , L. Ang , J. Liu , L. Cui , X. Song , S. Lv , E. Mahmud , JACC Cardiovasc. Interv. 2017, 10, 1307. 28624380 10.1016/j.jcin.2017.04.007

[12] J. Li , M. Guasch‐Ferre , W. Chung , M. Ruiz‐Canela , E. Toledo , D. Corella , S. N. Bhupathiraju , D. K. Tobias , F. K. Tabung , J. Hu , T. Zhao , C. Turman , Y. A. Feng , C. B. Clish , L. Mucci , A. H. Eliassen , K. H. Costenbader , E. W. Karlson , B. M. Wolpin , A. Ascherio , E. B. Rimm , J. E. Manson , L. Qi , M. A. Martinez‐Gonzalez , J. Salas‐Salvado , F. B. Hu , L. Liang , Eur. Heart J. 2020, 41, 2645. 32406924 10.1093/eurheartj/ehaa209 PMC7377580

[13] L. Huang , L. Wang , X. Hu , S. Chen , Y. Tao , H. Su , J. Yang , W. Xu , V. Vedarethinam , S. Wu , B. Liu , X. Wan , J. Lou , Q. Wang , K. Qian , Nat. Commun. 2020, 11, 3556. 32678093 10.1038/s41467-020-17347-6 PMC7366718

[14] A. Shahrjooihaghighi , H. Frigui , X. Zhang , X. Wei , B. Shi , A. Trabelsi , Proc IEEE Int. Symp. Signal Proc. Inf. Tech. 2017, 2017, 416. 30887013 10.1109/ISSPIT.2017.8388679 PMC6420823

[15] M. Y. Lee , T. Hu , Metabolites 2019, 9, 66.

[16] R. P. Grant , Clin. Chem. 2013, 59, 871. 23592506 10.1373/clinchem.2013.205435

[17] P. E. Geyer , L. M. Holdt , D. Teupser , M. Mann , Mol. Syst. Biol. 2017, 13, 942. 28951502 10.15252/msb.20156297 PMC5615924

[18] S. F. Weng , J. Reps , J. Kai , J. M. Garibaldi , N. Qureshi , PLoS One 2017, 12, e0174944. 28376093 10.1371/journal.pone.0174944 PMC5380334

[19] M. M. Gaschler , B. R. Stockwell , Biochem. Biophys. Res. Commun. 2017, 482, 419. 28212725 10.1016/j.bbrc.2016.10.086 PMC5319403

[20] E. A. Dennis , P. C. Norris , Nat. Rev. Immunol. 2015, 15, 511. 26139350 10.1038/nri3859 PMC4606863

[21] N. P. Paynter , R. Balasubramanian , F. Giulianini , D. D. Wang , L. F. Tinker , S. Gopal , A. A. Deik , K. Bullock , K. A. Pierce , J. Scott , M. A. Martinez‐Gonzalez , R. Estruch , J. E. Manson , N. R. Cook , C. M. Albert , C. B. Clish , K. M. Rexrode , Circulation 2018, 137, 841. 29459470 10.1161/CIRCULATIONAHA.117.029468 PMC5854187

[22] C. M. Manega , S. Fiorelli , B. Porro , L. Turnu , V. Cavalca , A. Bonomi , N. Cosentino , A. Di Minno , G. Marenzi , E. Tremoli , S. Eligini , Pharmacol. Res. 2019, 144, 336. 31028904 10.1016/j.phrs.2019.03.012

[23] J. Iqbal , M. T. Walsh , S. M. Hammad , M. M. Hussain , Trends Endocrinol. Metab. 2017, 28, 506. 28462811 10.1016/j.tem.2017.03.005 PMC5474131

[24] A. M. Poss , J. A. Maschek , J. E. Cox , B. J. Hauner , P. N. Hopkins , S. C. Hunt , W. L. Holland , S. A. Summers , M. C. Playdon , J. Clin. Invest. 2020, 130, 1363. 31743112 10.1172/JCI131838 PMC7269567

[25] D. D. Wang , E. Toledo , A. Hruby , B. A. Rosner , W. C. Willett , Q. Sun , C. Razquin , Y. Zheng , M. Ruiz‐Canela , M. Guasch‐Ferre , D. Corella , E. Gomez‐Gracia , M. Fiol , R. Estruch , E. Ros , J. Lapetra , M. Fito , F. Aros , L. Serra‐Majem , C. H. Lee , C. B. Clish , L. Liang , J. Salas‐Salvado , M. A. Martinez‐Gonzalez , F. B. Hu , Circulation 2017, 135, 2028. 28280233 10.1161/CIRCULATIONAHA.116.024261 PMC5496817

[26] R. N. Lemaitre , P. N. Jensen , A. Hoofnagle , B. McKnight , A. M. Fretts , I. B. King , D. S. Siscovick , B. M. Psaty , S. R. Heckbert , D. Mozaffarian , N. Sotoodehnia , Circ. Heart Fail 2019, 12, e005708. 31296099 10.1161/CIRCHEARTFAILURE.118.005708 PMC6629465

[27] P. N. Jensen , A. M. Fretts , A. N. Hoofnagle , C. M. Sitlani , B. McKnight , I. B. King , D. S. Siscovick , B. M. Psaty , S. R. Heckbert , D. Mozaffarian , N. Sotoodehnia , R. N. Lemaitre , J. Am. Heart Assoc. 2020, 9, e012853. 32019406 10.1161/JAHA.119.012853 PMC7070192

[28] R. Laaksonen , K. Ekroos , M. Sysi‐Aho , M. Hilvo , T. Vihervaara , D. Kauhanen , M. Suoniemi , R. Hurme , W. Marz , H. Scharnagl , T. Stojakovic , E. Vlachopoulou , M. L. Lokki , M. S. Nieminen , R. Klingenberg , C. M. Matter , T. Hornemann , P. Juni , N. Rodondi , L. Raber , S. Windecker , B. Gencer , E. R. Pedersen , G. S. Tell , O. Nygard , F. Mach , J. Sinisalo , T. F. Luscher , Eur. Heart J. 2016, 37, 1967. 27125947 10.1093/eurheartj/ehw148 PMC4929378

[29] Y. Deng , F. E. Rivera‐Molina , D. K. Toomre , C. G. Burd , Proc. Natl. Acad. Sci. U. S. A. 2016, 113, 6677. 27247384 10.1073/pnas.1602875113 PMC4914164

[30] J. D. Symons , E. D. Abel , Rev. Endocr. Metab. Disord. 2013, 14, 59. 23292334 10.1007/s11154-012-9235-3 PMC4180664

[31] P. P. Ruvolo , Leukemia 2001, 15, 1153. 11480555 10.1038/sj.leu.2402197

[32] Y. Yao , X. Yang , L. Sun , S. Sun , X. Huang , D. Zhou , T. Li , W. Zhang , N. A. Abumrad , X. Zhu , S. He , X. Su , EBioMedicine 2019, 41, 256. 30738828 10.1016/j.ebiom.2019.01.066 PMC6441949

[33] M. Makrecka‐Kuka , E. Sevostjanovs , K. Vilks , K. Volska , U. Antone , J. Kuka , E. Makarova , O. Pugovics , M. Dambrova , E. Liepinsh , Sci. Rep. 2017, 7, 17528. 29235526 10.1038/s41598-017-17797-x PMC5727517

[34] E. Strand , E. R. Pedersen , G. F. Svingen , T. Olsen , B. Bjorndal , T. Karlsson , J. Dierkes , P. R. Njolstad , G. Mellgren , G. S. Tell , R. K. Berge , A. Svardal , O. Nygard , J. Am. Heart Assoc. 2017, 6, e003620. 28159823 10.1161/JAHA.116.003620 PMC5523736

[35] T. Ahmad , J. P. Kelly , R. W. McGarrah , A. S. Hellkamp , M. Fiuzat , J. M. Testani , T. S. Wang , A. Verma , M. D. Samsky , M. P. Donahue , O. R. Ilkayeva , D. E. Bowles , C. B. Patel , C. A. Milano , J. G. Rogers , G. M. Felker , C. M. O'Connor , S. H. Shah , W. E. Kraus , J. Am. Coll. Cardiol. 2016, 67, 291. 26796394 10.1016/j.jacc.2015.10.079 PMC5429585

[36] A. Gulbins , F. Schumacher , K. A. Becker , B. Wilker , M. Soddemann , F. Boldrin , C. P. Muller , M. J. Edwards , M. Goodman , C. C. Caldwell , B. Kleuser , J. Kornhuber , I. Szabo , E. Gulbins , Mol. Psychiatry 2018, 23, 2324. 30038230 10.1038/s41380-018-0090-9 PMC6294742

[37] Z. X. Yuan , S. I. Rapoport , Prostaglandins Leukot. Essent. Fatty Acids 2015, 101, 9. 26234927 10.1016/j.plefa.2015.07.002 PMC4581970

[38] Y. Zhou , X. Tao , Z. Wang , L. Feng , L. Wang , X. Liu , R. Pan , Y. Liao , Q. Chang , Int. J. Mol. Sci. 2019, 20, 4282. 10.3390/ijms20174282 PMC6747550 31480539

[39] C. Pizzi , A. W. Rutjes , G. M. Costa , F. Fontana , A. Mezzetti , L. Manzoli , Am. J. Cardiol. 2011, 107, 972. 21256471 10.1016/j.amjcard.2010.11.017

[40] D. G. Altman , L. M. McShane , W. Sauerbrei , S. E. Taube , PLoS Med. 2012, 9, e1001216. 22675273 10.1371/journal.pmed.1001216 PMC3362085

[41] P. S. Chan , P. G. Jones , S. A. Arnold , J. A. Spertus , Circ. Cardiovasc. Qual. Outcomes 2014, 7, 640. 25185249 10.1161/CIRCOUTCOMES.114.000967 PMC4282595

[42] K. Li , X. Wang , V. R. Pidatala , C. P. Chang , X. Cao , J. Proteome Res. 2014, 13, 5879. 25327737 10.1021/pr5007813

[43] M. Yuan , S. B. Breitkopf , X. Yang , J. M. Asara , Nat. Protoc. 2012, 7, 872. 22498707 10.1038/nprot.2012.024 PMC3685491

[44] G. Nyamundanda , I. C. Gormley , Y. Fan , W. M. Gallagher , L. Brennan , BMC Bioinformatics 2013, 14, 338. 24261687 10.1186/1471-2105-14-338 PMC4222287

[45] Z. Pang , J. Chong , S. Li , J. Xia , Metabolites 2020, 10, 186. 10.3390/metabo10050186 PMC7281575 32392884

[46] J. Xia , D. I. Broadhurst , M. Wilson , D. S. Wishart , Metabolomics 2013, 9, 280. 23543913 10.1007/s11306-012-0482-9 PMC3608878

