# Supplementary material for: Machine Learning Identifies Metabolic Signatures that Predict the Risk of Recurrent Angina in Remitted Patients after Percutaneous Coronary Intervention: A Multicenter Prospective Cohort Study
Source: Adv Sci (Weinh). 2021 Mar 8;8(10):2003893. doi: 10.1002/advs.202003893 (PMC8132066; doi:10.1002/advs.202003893)
Supplement: Supplementary file 1 — Supporting Information [file ADVS-8-2003893-s001.pdf]

© 2021 Wiley-VCH GmbH

ADVANCED SCIENCE
Open Access

Supporting Information

for *Adv. Sci.,* DOI: 10.1002/advs.202003893

Machine learning identifies metabolic signatures that predict the risk of recurrent angina in remitted patients after percutaneous coronary intervention: A multicenter prospective cohort study

Song Cui, Li Li, Yongjiang Zhang, Jianwei Lu, Xiuzhen Wang, Xiantao Song, Jinghua Liu, Kefeng Li**

Supporting Information

Machine learning identifies metabolic signatures that predict the risk of recurrent angina in remitted patients after percutaneous coronary intervention: A multicenter prospective cohort study

Song Cui, Li Li, Yongjiang Zhang, Jianwei Lu, Xiuzhen Wang, Xiantao Song, Jinghua Liu, Kefeng Li**

Table of content

Supplemental Methods....................4

Supplemental Results 8

Table S1 Characteristics of patients in the additional discovery cohort (n = 775)....................9

Table S2. Characteristics of patients in the external validation cohort from another hospital (n = 130) 10

Table S3. Metabolomics quality control: reproducibility of metabolite quantification. 11

Table S4. List of metabolites ranked by VIP scores that contributes to the separation of angina recurrence from angina-free in the discovery cohort. 12

Table S5. List of metabolites ranked by VIP scores that contributes to the separation of angina recurrence from angina-free in the additional discovery cohort....................15

Table S6. The associations between the baseline characteristics and future angina recurrence in the discovery cohort. 18

Table S7. Associations of individual metabolites with the risk of future angina recurrence in the discovery cohort. 19

Table S8. List of top 60 metabolites ranked by their contributions to classification accuracy of angina recurrence in random forest algorism in the discovery cohort. 21

Table S9. List of top 60 metabolites ranked by their contributions to classification accuracy of angina recurrence in random forest algorism in the additional discovery cohort....................23

Table S10. The MRM transitions, compound-dependent parameters, and their internal standards for the analysis of the metabolic predictors using stable isotope-dilution LC-MS/MS....................25

Table S11. The coefficients of 6 selected metabolic predictors in the multivariate logistic regression model of external validation cohort. 25

Figure S1. The representative images of the coronary angiography before and after the percutaneous coronary intervention (PCI) for a patient with stable angina....................26

Figure S2. The representative chromatogram for the metabolomic analysis. 27

Figure S3. Partial least square discriminant analysis (PLS-DA) revealed the clear separation of the metabolic profiles in the plasma of remitted patients with angina recurrence within nine months after PCI from those of angina-free patients in the additional discovery cohort....................28

Figure S4. Assessment of partial least square discriminant analysis (PLS-DA) model for discriminating remitted patients with future angina recurrence from angina-free patients in the discovery cohort....................29

Figure S5. Assessment of the PLS-DA model for discriminating remitted patients with future angina recurrence from angina-free patients in the additional discovery cohort 30

Figure S6. The top 25 discriminating metabolites in the plasma samples of remitted patients between recurrent angina and angina-free revealed by variable importance in projection (VIP) scores in the additional discovery cohort. 31

Figure S7. The top 15 metabolites ranked by their contributions to classification accuracy....................32

Figure S8. The prediction performance for future angina recurrence in the discovery cohort (n = 750) using the features selected by each individual machine learning algorithm. 33

Figure S9. The representative LC-MS/MS chromatograms of metabolic predictors in a sample of the external validation cohort....................34

Figure S10. The standard curves and linear range for the selected metabolite predictors in the plasma matrix. 35

Supplemental Methods

Study population

The inclusion criteria were hospitalized CAD patients with stable angina. All patients had at least one lesion, and each patient received one second-generation rapamycin-eluting stent (Firebird 2, Microport, Shanghai, China) (Figure S1). In addition to percutaneous coronary intervention (PCI), patients were given optimal medical therapy (OMT). The symptom of angina was dramatically alleviated before the patients were discharged from the hospital. The exclusion criteria were as follows: patient with the lesion of > 90% stenosis or total occlusion, active inflammation (High sensitive C-reactive protein [hsCRP] >1.0 mg/dl), renal disease (serum creatinine > 3.0 mg/dl or ongoing hemodialysis), the onset of cancer in the previous five years, coronary artery bypass grafting, contraindications to iodine media, and severe hepatic insufficiency.

Finally, three cohorts were obtained in this study. Total 750 patients in Beijing Anzhen Hospital were enrolled between December 2015 and May 2016 as the discovery cohort (cohort 1) and another 775 patients from February 2017 to August 2017 for additional independent discovery cohort (cohort 2). The external validation cohort (cohort 3) was recruited in Qufu Hospital, Shandong Province from January 2018 through June 2018. The written informed consent was obtained from all subjects. The patient characteristics are listed in Table 1 (Discovery cohort), Table S1 (Additional discovery cohort), and Table S2 (External validation cohort).

Blood collection and follow-up for angina recurrence

After an overnight fast, venous blood was collected from each patient at 48 h after PCI. Plasma was separated by centrifugation at 900 g for 10 minutes at room temperature within one hour of collection. The resulting fresh lithium-heparin plasma was transferred to new tubes and stored at -80 ˚C for further analysis.

The patients were discharged after the blood draw. They were followed up every 30 days for angina recurrence up to 270 days (9 months). Seattle angina questionnaire-7 (SAQ-7) is a validated, self-administered, disease-specific measure for patients with CAD to evaluate the severity of angina. The SAQ-7 was conducted at each follow-up for all patients.

Extraction of metabolites from plasma for metabolomic analysis

Ninety-five (95) µL of plasma was thawed, mixed with 5 µL of custom-synthesized stable isotope standards and extracted with four volumes (400 µL) of prechilled (-20 ˚C) extraction buffer containing acetonitrile and methanol (50: 50, v/v). The mixture was incubated on crushed ice for 10 min and then centrifuged at 16,000 g for 10 min at 4 ˚C. The supernatant containing the extracted metabolites was transferred to labeled cryotubes and stored at -80 ˚C for metabolomic analysis.

Metabolomic analysis

Metabolomic analysis was performed on a Shimadzu LC-20A ultra-high-performance liquid chromatography coupled with SCIEX QTRAP 6500 triple quadrupole mass spectrometer (LC-MS/MS) using a broad-spectrum targeted metabolomic approach. A total of 606 metabolites covering the major metabolic pathways were targeted by scheduled multiple reaction monitoring (sMRM) under Analyst v1.6.2 software control in both negative and positive mode with rapid polarity switching (20 ms). Nitrogen was used for curtain gas (set to 30), collision gas (set to high), ion source gas 1 and 2 (set to 35). The source temperature was set to 500 ˚C. Spray voltage

was set to -4500 V for negative mode and 5500 V for positive mode. Compound-dependent MRM parameters were optimized using the purified standards. Ten µL of the extract was injected into a 250 mm × 2.0 mm, 4 µm polymer-based NH_2 HPLC column (Asahipak NH_2P-40 2E, Showa Denko America, Inc., NY) held at 25 °C for chromatographic separation. The mobile phase was solvent A: 95% water, 5% acetonitrile with 20 mM NH_4OAC, and 20 mM NH_4OH (pH 9.4); solvent B: 100% acetonitrile. Separation was achieved using the following gradient: 0-3.5 min: 95% B, 3.6-8 min: 85% B, 8.1-13 min: 75% B, 14-30 min: 0% B, 31-41 min: 95% B, 41.1 min: end. The flow rate was 250 µL/min. The chromatographic peaks were inspected and integrated using MultiQuant 3.0 (SCIEX, USA).

The pooled plasma samples were used as quality control (QC) and injected four times in each batch. Metabolites with the inter-day coefficient of variations (CVs) > 25% in QC samples were excluded.

External validation using the patients from another hospital and a different analytical approach

To validate the selected metabolite biomarkers for the prediction of future angina recurrence, we quantified these six metabolites in the external validation cohort (cohort 3) plasma samples by stable-isotope dilution LC-MS/MS. Briefly, 95 µL of plasma was thoroughly mixed with 5 µL of stable isotope internal standard mix containing palmitoylcarnitine (N-methyl-D3) (Catalog No. NSK-B, Cambridge Isotopes), 12-HETE-d8 (Catalog No. 334570, Cayman Chemicals), PC(16:0/16:0)-d62 (Catalog No. DLM-606, Cambridge Isotopes), PI (17:0/20:4) (Catalog No. lm1502, Avanti Polar Lipids), SM (d18:1/18:1-d9) (Catalog No. 791649, Avanti Polar Lipids) and Cer(d18:1/18:1)-$^{13}C_{18}$. The extraction method was the same as described in the metabolomic analysis section.

Quantitative analysis of the target compounds was performed on a Shimadzu LC-20A UHPLC system coupling with a QTRAP 6500 triple quadrupole mass spectrometer (SCIEX, USA). Ten μL of the extract was loaded onto ACQUITY UPLC BEH C_{18} column (1.7 μm particle size, 2.1 × 150 mm, Waters) held at 35 ºC for separation. Solvent A consisted of water/IPA/methanol (90:5:5, v/v/v) with 20 mM NH_4OAC and solvent B was isopropanol/ methanol (50:50, v/v/) with 20 mM NH_4OAC. The flow rate was 0.3 mL/min. The analytes were separated using linear gradient elution to 100% B over 25 min and isocratic 100% B for 4 min. The chromatographic system was returned to the initial conditions in 1 min, followed by a 2-min equilibration before the subsequent injection.

MS/MS analysis was performed in both negative and positive model with rapid polarity switch (3 ms). The ion spray voltage was -4500 V for negative mode and 5500 V for positive mode. The source temperature was set at 500 ºC. The MRM transitions were optimized using the purified standards, and two transitions were developed for each analyte.

The matrix-specific calibration curves for the targeted analytes were generated by plotting the peak area ratios (analyte/internal standard) versus the concentration ratios (analyte/internal standard) and fitting to linear regression.

Supplemental Results

Metabolomic method reproducibility

Out of 606 targeted metabolites, 458 metabolites were detected in all the samples without missing values. Forty-eight (48) metabolites with inter-day CVs >25% were excluded, and 407 metabolites were finally included in the analysis. The representative chromatogram is shown in Figure S2. The intra-day and inter-day batch correlation r values were 0.995 and 0.981, respectively, for theses metabolites in QC samples. The median intra-day and inter-day coefficient of variation (CVs) were 10.2% and 11.5%, respectively, for QC samples (Table S3). These metrics suggested excellent reproducibility for the included metabolites.

Method validation for quantification of metabolite predictors using stable-isotope dilution LC-MS/MS

The optimal compound-dependent parameters for LC-MS/MS analysis of six metabolic predictors are described in Table S10. The representative chromatogram is shown in Figure S9. All six metabolites were chromatographically separated with the optimized gradient on an ACQUITY UPLC BEH C_{18} column. Figure S10 shows the standard curves and linear ranges for the quantification of these metabolites. The method is reproducible with the median intra-day CV of 2.14% and inter-day CV of 6.73%, which are below the acceptance limit (20% CV) of FDA's guideline for bioanalytical method validation using LC-MS.

Table S1 Characteristics of patients in the additional discovery cohort (n = 775)

Characteristics	Patients with recurrent angina in remission (n = 198)	Angina-free (n = 577)	*P* value (Angina vs. Angina-free)	Hedges' g effect size
Male (%)	98 (49.5%)	287 (49.7%)	0.89	0.0049
Age (years)	59 ± 9	61 ± 11	0.26	0.19
BMI (kg/m2)	23.3 ± 3.8	22.8 ± 4.2	0.15	0.12
Smoking (n, %)	58 (29.3%)	191 (33.1%)	0.28	0.082
Hypertension	43 (21.7%)	111 (19.2%)	0.51	0.061
LDL-C (mg/dl)	87 ± 11.2	89 ± 14.1	0.073	0.15
HDL-C (mg/dl)	43 ± 5.9	42 ± 7.2	0.08	0.15
Uric acid (μmol/L)	252 ± 112	247 ± 96	0.55	0.049
HbA1c (%)	5.9 ± 2.3	5.7 ± 1.8	0.22	0.11
hsCRP (mg/dl)	0.13 (0.06 – 0.25)	0.15 (0.04 – 0.26)	0.32	0.053
Stent diameter (mm)	2.5 ± 0.78	2.4 ± 0.64	0.09	0.14
Ejection fraction (%)	67 ± 13.8	65 ± 12.4	0.06	0.16
Medications				
Aspirin	198 (100%)	577 (100%)	1.00	0.00
Clopidogrel	198 (100%)	577 (100%)	1.00	0.00
ACEI/ARB	46 (23.2%)	140 (24.3%)	0.70	0.024
β-blocker	52 (26.3%)	157 (27.2%)	0.78	0.015
Statin	63 (31.5%)	166 (28.7%)	0.47	0.067

Notes: Values are mean ± SD, n (%), or median (interquartile range). Differences between recurrent angina and angina-free groups were analyzed using the Student's t-test (parametric distribution), Mann-Whitney U test (nonparametric distribution), or two-proportion z-test (Categorical or proportional data). The effect size between two groups was calculated by Hedge’s statistic.

Table S2. Characteristics of patients in the external validation cohort from another hospital (n = 130)

**Characteristics**	**Patients with recurrent angina in remission**	**Angina-free**	***P* value (Angina vs. Angina-free)**	**Hedges' g effect size**
Total number (Male %)	40 (45.2%)	90 (52.4%)	0.34	0.14
Age (years)	63 ± 11	59 ± 12	0.12	0.34
BMI (kg/m2)	23 ± 4.1	21 ± 5.4	0.07	0.39
Smoking (n, %)	9 (22.6%)	20 (22.2%)	0.85	0.0067
Hypertension	8 (19.4%)	16 (17.5%)	0.95	0.057
LDL-C (mg/dl)	94 ± 2.7	95 ± 3.8	0.14	0.29
HDL-C (mg/dl)	43 ± 3.8	44 ± 5.2	0.27	0.21
Uric acid (μmol/L)	257 ± 89	249 ± 93	0.65	0.087
HbA1c (%)	5.9 ± 0.65	5.7 ± 0.77	0.15	0.27
hsCRP (mg/dl)	0.16 (0.04 – 0.21)	0.14 (0.03 – 0.19)	0.075	0.39
Stent diameter (mm)	2.7 ± 0.35	2.6 ± 0.46	0.22	0.23
Ejection fraction (%)	65 ± 8.8	62 ± 9.4	0.14	0.33
**Medications**				
Aspirin	40 (100%)	90 (100%)	1.00	0.00
Clopidogrel	40 (100%)	90 (100%)	1.00	0.00
ACEI/ARB	9 (22.6%)	19 (20.9%)	0.96	0.034
β-blocker	11 (25.8%)	20 (22.2%)	0.67	0.12
Statin	12 (29.1%)	26 (28.5%)	0.94	0.024

Notes: Values are mean ± SD, n (%), or median (interquartile range). Differences between angina and angina-free groups were analyzed using Student's t-test (parametric distribution), Mann-Whitney U test (nonparametric distribution), or two-proportion z-test (Categorical or proportional data). The effect size between the two groups was calculated by Hedge's statistic.

Table S3. Metabolomics quality control: reproducibility of metabolite quantification.

QC samples	Intra-day Pearson's correlation coefficient r	Inter-day Pearson's correlation coefficient r	Intra-day median CV	Inter-day median CV
407 metabolites detected in QC samples	0.995	0.981	10.2%	11.5%

Notes: QC samples were prepared by mixing all the samples (recurrent angina and angina-free) in the cohort. QC samples were run four times each day. QC: Quality control; CV: Coefficient of variation.

Table S4. List of metabolites ranked by VIP scores that contributes to the separation of angina recurrence from angina-free in the discovery cohort.

Metabolites	Comp. 1	Comp. 2	Comp. 3	Comp. 4	Comp. 5
LysoPC(18:0)	2.3386	2.299	2.2909	2.2857	2.2826
Phenyllactic acid	2.329	2.2936	2.2838	2.2819	2.278
PI(36:2)	2.2899	2.2503	2.2401	2.2357	2.232
L-Palmitoylcarnitine	2.2884	2.2742	2.2638	2.2589	2.255
Ethanolamine	2.2081	2.1742	2.1715	2.1676	2.1639
Uridine	2.1627	2.1346	2.1284	2.124	2.1203
Myristoylcarnitine	2.1614	2.1309	2.121	2.1175	2.1145
CL(18:2/18:2/18:2/20:4)	2.1077	2.072	2.0626	2.0598	2.0564
Isovalerylglycine	2.105	2.0724	2.0633	2.0587	2.0551
12-HETE	2.0532	2.0255	2.016	2.0115	2.0081
Octadecanoylcarnitine	1.9918	1.9747	1.9664	1.9627	1.9595
SM(d18:1/16:2)	1.9824	1.9484	1.9396	1.9372	1.9343
SM(d18:1/18:1)	1.9559	1.9288	1.9224	1.9185	1.9154
Pseudouridine	1.9353	1.9056	1.8976	1.8934	1.8903
Indoxyl sulfate	1.9187	1.9022	1.8955	1.8914	1.8883
SM(d18:1/16:0)	1.8998	1.8741	1.8662	1.8638	1.8608
cis-aconitic acid	1.8937	1.8632	1.8582	1.8543	1.8511
Carnosine	1.8933	1.8621	1.8581	1.8543	1.851
4-Hydroxyphenyllactic acid	1.8898	1.8589	1.8502	1.8488	1.8457
L-Glutamic acid	1.8344	1.8181	1.8097	1.8063	1.8049
4-Hydroxyphenylpyruvic acid	1.8247	1.7953	1.7872	1.7832	1.7801
7-Dehydrocholesterol	1.8218	1.7984	1.7929	1.7889	1.787
Ceramide(d18:1/18:2 OH)	1.8034	1.781	1.7743	1.7711	1.768
SM(d18:1/16:0 OH)	1.7955	1.7749	1.7666	1.7627	1.7596
SM(d18:1/26:0)	1.7823	1.755	1.7519	1.7524	1.7497
CL(18:2/18:2/18:1/18:1)	1.7722	1.7511	1.743	1.7392	1.739
L-Valine	1.7685	1.7422	1.7384	1.7346	1.7318
Adipic acid	1.7658	1.7361	1.7281	1.7258	1.7272
Oleic acid	1.7649	1.7376	1.7309	1.7277	1.7264
5-HETE	1.7311	1.7238	1.7177	1.7143	1.7151
Niacinamide	1.731	1.7101	1.7028	1.6996	1.6972
PI(36:1)	1.6828	1.6641	1.6563	1.6545	1.652
PS(34:1)	1.6626	1.6633	1.6573	1.6536	1.651
11-HETE	1.6485	1.6362	1.6314	1.6279	1.6254
2-Hydroxy-3-methylbutyric acid	1.6462	1.6178	1.6115	1.6081	1.6063
PI(36:0)	1.6374	1.6304	1.6252	1.6247	1.6231
Ceramide(d18:1/16:0)	1.6312	1.6103	1.6045	1.601	1.5982
Ceramide(d18:1/12:0)	1.6286	1.6087	1.6012	1.5978	1.5966
Azelaic acid	1.6217	1.5947	1.5876	1.5867	1.59
2-Hydroxyglutarate	1.5839	1.5567	1.5494	1.5472	1.5456

Hydroxyisocaproic acid	1.5794	1.5522	1.5451	1.5457	1.543
Homoarginine	1.5772	1.557	1.5497	1.5509	1.5483
SM(d18:1/16:1)	1.5723	1.5626	1.5618	1.5583	1.5558
Sphingosine 1-phosphate	1.5662	1.5404	1.54	1.5381	1.5354
CL(18:2/18:2/18:2/16:1)	1.5644	1.5396	1.5346	1.5317	1.5292
SM(d18:1/16:2 OH)	1.5614	1.5483	1.5415	1.5384	1.5362
N-acetylserine	1.5607	1.5348	1.5297	1.5264	1.5246
S-Adenosylhomocysteine	1.5406	1.5247	1.5209	1.5175	1.5164
Betaine	1.5392	1.5335	1.5337	1.5302	1.5292
PC(18:1/22:6)	1.5306	1.5042	1.5026	1.5015	1.4989
SM(d18:1/20:0 OH)	1.512	1.5114	1.5052	1.5025	1.5
9-Hexadecenoylcarnitine	1.4892	1.4655	1.4637	1.4606	1.4585
Dopamine	1.4861	1.4794	1.4743	1.471	1.4685
PA(18:0/18:1)	1.4726	1.4722	1.4657	1.4624	1.4601
SM(d18:1/16:1 OH)	1.4708	1.4564	1.452	1.4499	1.4482
PC(18:0/18:2)	1.4336	1.4183	1.4127	1.4143	1.4124
Phenylacetylglutamine	1.4302	1.406	1.4026	1.4011	1.3991
SM(d18:1/22:0 OH)	1.4299	1.4093	1.4029	1.4003	1.4013
2-Ketohexanoic acid	1.4289	1.4043	1.3979	1.3964	1.3945
Itaconic acid	1.4269	1.4024	1.3963	1.3932	1.393
Tetranor-PGEM	1.4268	1.4031	1.3967	1.396	1.3939
BMP(18:1/16:1)	1.4249	1.4056	1.4046	1.4026	1.4002
SM(d18:1/26:1)	1.4225	1.4069	1.4006	1.399	1.398
Hippuric acid	1.4222	1.4057	1.4055	1.403	1.402
Suberic Acid	1.42	1.3954	1.3891	1.3894	1.3898
CL(18:2/18:2/18:2/18:2)	1.4117	1.3901	1.384	1.3823	1.3801
L-Tyrosine	1.4063	1.3828	1.3764	1.3734	1.3748
2-Isopropylmalic acid	1.4034	1.3792	1.3743	1.3713	1.3703
L-Acetylcarnitine	1.4019	1.3834	1.3798	1.3779	1.3762
PI(38:5)	1.3935	1.3772	1.3708	1.3681	1.3678
PC(36:1)	1.3886	1.3796	1.3732	1.3746	1.3744
SM(d18:1/18:1 OH)	1.3882	1.3755	1.3696	1.368	1.366
Pyruvic acid	1.3829	1.3682	1.3639	1.3651	1.3629
Ribose 5-phosphate	1.3828	1.3783	1.3724	1.3747	1.3735
SM(d18:1/18:2)	1.3769	1.3542	1.3484	1.351	1.3487
Adipoylcarnitine	1.3764	1.3527	1.3558	1.3529	1.3508
BMP(18:1/18:2)	1.374	1.3603	1.3673	1.3662	1.3669
PS(34:2)	1.3686	1.3788	1.378	1.3752	1.3729
THC(18:1/24:1)	1.3609	1.3434	1.3441	1.3425	1.3425
11,12-DiHETrE	1.3591	1.3409	1.3355	1.3325	1.3397
N-acetylputrescine	1.355	1.3399	1.3417	1.3397	1.338
THC 18:1/22:0	1.3474	1.3405	1.3373	1.3345	1.3379
Tiglylglycine	1.3388	1.3159	1.3101	1.3075	1.3062
7-Methylguanosine	1.3302	1.3119	1.3082	1.307	1.3047

Homocitrulline	1.3297	1.3157	1.3096	1.3067	1.3047
N-oleoylethanolamine	1.3279	1.3061	1.311	1.3084	1.3075
Glucosamine	1.3091	1.2922	1.2867	1.2837	1.2844
DHC(18:1/16:0)	1.3017	1.3202	1.3141	1.3112	1.3091
4-Hydroxybenzoic acid	1.2979	1.2773	1.2745	1.2717	1.2705
Ceramide(d18:1/16:1 OH)	1.2931	1.3008	1.2948	1.2919	1.2913
MHC(18:1/16:0)	1.2918	1.2909	1.2854	1.2834	1.2821
Glycerol	1.2792	1.2669	1.2621	1.2592	1.2593
PI(38:3)	1.2735	1.2986	1.2925	1.2897	1.2875
Taurine	1.2701	1.2619	1.2618	1.2631	1.2611
5-Methylthioadenosine	1.2642	1.2638	1.2621	1.2594	1.2578
Shikimate-3-phosphate	1.2606	1.2395	1.2341	1.2317	1.2297
Allantoic acid	1.2581	1.2424	1.2391	1.2379	1.2397
Inosine	1.2552	1.247	1.2412	1.2384	1.2371
Creatine	1.2534	1.2382	1.2332	1.2305	1.2329
2-Octenoylcarnitine	1.2525	1.237	1.2323	1.231	1.2301
Butyrylcarnitine	1.2508	1.2296	1.2249	1.2235	1.2223
THC18:1/16:0	1.2495	1.2325	1.2409	1.2381	1.2407
Xanthine	1.2489	1.2407	1.2372	1.2355	1.235
PE(34:1)	1.2476	1.2676	1.2637	1.261	1.262
SM(d18:1/18:0)	1.2426	1.251	1.2457	1.2472	1.2451
Desmosterol	1.23	1.2167	1.213	1.2107	1.2089
L-Kynurenine	1.2282	1.2138	1.2094	1.2094	1.2073
Quinolinic Acid	1.2229	1.202	1.1964	1.1941	1.1954
Palmitoylethanolamide	1.2032	1.1931	1.1918	1.1902	1.1891
Retinol	1.2019	1.1822	1.1831	1.1832	1.1904
Gluconic acid	1.2005	1.1926	1.188	1.1875	1.1856

Note: VIP ⩾ 1.2 was considered as statistically significant.

Table S5. List of metabolites ranked by VIP scores that contributes to the separation of angina recurrence from angina-free in the additional discovery cohort.

Metabolites	Comp. 1	Comp. 2	Comp. 3	Comp. 4	Comp. 5
LysoPC(18:0)	3.2255	3.2247	3.2244	3.2243	3.2242
12-HETE	2.5357	2.5483	2.5522	2.5528	2.5533
Isovalerylglycine	2.4851	2.4846	2.4843	2.4842	2.4841
Azelaic acid	2.4128	2.4122	2.4119	2.4118	2.4117
Niacinamide	2.4023	2.4017	2.4015	2.4015	2.4014
PI(36:2)	2.2972	2.2967	2.2967	2.2966	2.2965
Quinolinic Acid	2.1521	2.1516	2.1514	2.1513	2.1513
Myristoylcarnitine	2.0607	2.0702	2.0718	2.0718	2.0725
Retinol	2.0575	2.057	2.0568	2.0569	2.0569
Adipic acid	2.0074	2.0069	2.0067	2.0067	2.0066
L-Palmitoylcarnitine	1.9679	1.9675	1.9673	1.9673	1.9672
4-Hydroxyphenylpyruvic acid	1.9197	1.9193	1.9192	1.9191	1.9191
PE(34:1)	1.9078	1.9073	1.9071	1.907	1.907
Hippuric acid	1.8125	1.8121	1.8119	1.8119	1.8118
L-Glutamic acid	1.8074	1.8071	1.8069	1.8069	1.8068
Cytidine	1.7982	1.7978	1.7977	1.7976	1.7976
SM(d18:1/16:2)	1.7978	1.7975	1.7973	1.7972	1.7972
S-Adenosylhomocysteine	1.7732	1.7727	1.7726	1.7725	1.7724
Biotin	1.7301	1.73	1.7303	1.7302	1.7302
Phenyllactic acid	1.7099	1.7095	1.7094	1.7093	1.7093
Dopamine	1.6771	1.6768	1.6767	1.6766	1.6766
Hexose Disaccharide Pool	1.6732	1.6731	1.673	1.6732	1.6731
Cholic acid	1.6534	1.6547	1.656	1.656	1.6561
Anandamide	1.6404	1.6401	1.6401	1.64	1.64
2-Ketohexanoic acid	1.6334	1.6331	1.6329	1.6329	1.6328
2-Hydroxyglutarate	1.6327	1.6324	1.6322	1.6322	1.6322
Oleic acid	1.6252	1.6248	1.6247	1.6246	1.6246
Indoxyl sulfate	1.6004	1.6002	1.6002	1.6002	1.6001
PI(36:0)	1.5967	1.5966	1.5965	1.5965	1.5965
11-HETE	1.5946	1.5943	1.5941	1.5941	1.594
CL(18:2/18:2/18:2/20:4)	1.5938	1.5935	1.5933	1.5933	1.5933
Phenylacetylglutamine	1.5913	1.5912	1.5912	1.5912	1.5912
Testosterone	1.5873	1.5869	1.587	1.587	1.587
Carnosine	1.587	1.5868	1.5867	1.5866	1.5866
Adipoylcarnitine	1.585	1.5846	1.5845	1.5844	1.5844
Ceramide(d18:1/16:0)	1.566	1.5658	1.5657	1.5656	1.5656
Ceramide(d18:1/18:2 OH)	1.5572	1.5585	1.5585	1.5596	1.561
PI(36:1)	1.5472	1.5468	1.5467	1.5466	1.5466
2-Octenoylcarnitine	1.5472	1.5469	1.5468	1.5467	1.5467
Deoxyadenosine	1.5471	1.5468	1.5467	1.5468	1.5468

PA(16:1/16:1)	1.5465	1.5461	1.546	1.5459	1.5459
THC 18:1/22:0	1.516	1.5157	1.5156	1.5156	1.5156
Glycerol	1.5106	1.5105	1.5104	1.5103	1.5103
Methylguanidine	1.5071	1.507	1.5069	1.5068	1.5068
cis-aconitic acid	1.5034	1.5031	1.503	1.5029	1.5029
Itaconic acid	1.4895	1.4892	1.489	1.4889	1.4889
2-Hydroxy-3-methylbutyric acid	1.4818	1.4814	1.4813	1.4812	1.4812
PE(32:2)	1.4813	1.4809	1.4808	1.4807	1.4807
9-Hexadecenoylcarnitine	1.4725	1.4723	1.4722	1.4722	1.4722
Sphingosine 1-phosphate	1.4713	1.471	1.4709	1.4708	1.4708
Suberic acid	1.4534	1.4549	1.4548	1.4549	1.4549
THC 18:1/16:0	1.4323	1.4321	1.432	1.432	1.4319
4-Hydroxyphenyllactic acid	1.4193	1.4191	1.419	1.419	1.419
Deoxyadenosine monophosphate	1.4185	1.4182	1.4181	1.418	1.418
Uridine	1.4174	1.4172	1.4171	1.4171	1.417
THC 18:1/24:1	1.4154	1.4151	1.4149	1.4148	1.4148
Inosine	1.4108	1.4104	1.4103	1.4102	1.4102
7-Dehydrocholesterol	1.3893	1.389	1.3888	1.3888	1.3887
5-HETE	1.3668	1.3665	1.3663	1.3663	1.3663
SM(d18:1/18:1)	1.3656	1.3653	1.3653	1.3652	1.3652
Ketoleucine	1.3646	1.3642	1.3641	1.364	1.364
Tiglylglycine	1.3625	1.3623	1.3622	1.3621	1.3621
Deoxycholic acid	1.3538	1.3544	1.3546	1.3547	1.3548
Ceramide(d18:1/12:0)	1.3531	1.3527	1.3526	1.3526	1.3525
Ceramide(d18:1/16:1 OH)	1.346	1.3457	1.3455	1.3455	1.3454
SM(d18:1/16:0)	1.3455	1.3453	1.3452	1.3451	1.3451
PI(16:0/16:0)	1.3416	1.3413	1.3412	1.3413	1.3412
Butyrylcarnitine	1.3401	1.3397	1.3396	1.3396	1.3395
MHC(18:1/22:0)	1.3332	1.333	1.333	1.333	1.3329
2-Isopropylmalic acid	1.329	1.3287	1.3286	1.3285	1.3285
PI(36:4)	1.3225	1.3223	1.3221	1.3221	1.3221
PE(36:4)	1.3103	1.31	1.3099	1.3099	1.3098
Nicotinamide N-oxide	1.306	1.3057	1.3056	1.3056	1.3056
Methylcysteine	1.2964	1.2961	1.296	1.2959	1.2959
MHC(18:2/16:0)	1.2901	1.2898	1.2897	1.2897	1.2897
Histamine	1.2886	1.2883	1.2882	1.2882	1.2882
N-oleoylethanolamine	1.2791	1.2788	1.2786	1.2786	1.2786
PE(36:2)	1.2655	1.2651	1.265	1.265	1.2649
Malonic acid	1.2651	1.2648	1.2646	1.2646	1.2645
Homoarginine	1.2615	1.2614	1.2614	1.2613	1.2613
Octadecanoylcarnitine	1.2508	1.2505	1.2504	1.2504	1.2504
L-Aspartic acid	1.2438	1.2435	1.2433	1.2433	1.2433
DHC(18:1/16:0)	1.2379	1.2376	1.2375	1.2374	1.2374
BMP(18:1/16:1)	1.2367	1.2365	1.2364	1.2363	1.2363

2-Pyrocatechuic acid	1.2346	1.2344	1.2345	1.2344	1.2344
Desmosterol	1.2302	1.23	1.2299	1.2299	1.2298
Homocitrulline	1.219	1.2189	1.219	1.2189	1.2189
PG(34:1)	1.2188	1.2186	1.2186	1.2185	1.2185
Hypoxanthine	1.2103	1.2102	1.2101	1.2101	1.21
Ceramide(d18:1/24:2 OH)	1.2096	1.2093	1.2092	1.2092	1.2092
BMP(18:1/18:2)	1.203	1.2028	1.2028	1.2028	1.2027

Note: VIP ⩾ 1.2 was considered as significant.

Table S6. The associations between the baseline characteristics and future angina recurrence in the discovery cohort.

Baseline variables	Univariate model		Multivariate model	
	Hazard ratio (95% CI)	*P* value	Hazard ratio (95% CI)	*P* value
Age at blood draw				
>= 65	1.15 (1.03-1.27)	0.023	1.23 (1.11-1.35)	0.034
<65	1.07 (0.87-1.33)	0.23	1.03 (0.91-1.17)	0.19
Male sex	0.80 (0.71-1.04)	0.072	0.85 (0.72-0.97)	0.075
BMI				
> 18.5 & < 25	0.73 (0.56-1.19)	0.21	0.79 (0.64-1.25)	0.26
>= 25 & < 30	0.91 (0.76-1.46)	0.25	1.17 (0.85-1.34)	0.32
>= 30	1.12 (1.01-1.24)	0.036	1.21 (1.09-1.32)	0.016
Hypertension				
No	0.77 (0.65-0.93)	0.0061	0.69 (0.57-0.84)	0.0043
Yes	1.23 (1.05-1.45)	0.012	1.26 (1.06-1.49)	0.0065
Smoking				
Never	0.91 (0.85-1.23)	0.54	0.86 (0.73-1.35)	0.46
Former	1.03 (0.91-1.35)	0.33	1.07 (0.89-1.26)	0.38
Current	1.11 (0.98-1.42)	0.062	1.16 (0.92-1.37)	0.13
LDL (mg/dl)				
>=100	1.15 (1.06-1.24)	0.016	1.08 (1.06-1.24)	0.028
<100	1.07 (0.79-1.19)	0.17	0.86 (0.69-1.05)	0.41
hsCRP	1.38 (1.17-1.63)	0.0065	1.42 (1.19-1.69)	0.0011
Statin Use				
No	1.06 (0.87-1.21)	0.055	0.98 (0.83-1.14)	0.058
Yes	1.12 (0.93-1.25)	0.073	1.34 (0.87-1.42)	0.16

Notes: Multivariate Cox regression model was adjusted using all the variables. CI: Confidence interval; BMI: Body mass index; hsCRP: High sensitivity C-reactive protein; LDL: Low density lipoprotein.

Table S7. Associations of individual metabolites with the risk of future angina recurrence in the discovery cohort.

Metabolites	Odds ratio	95% CI	Adjusted *P* value
12-HETE	2.92	2.52 - 3.09	0.0055
L-Palmitoylcarnitine	2.05	1.89 - 2.23	0.0084
Myristoylcarnitine	1.86	1.64 - 2.01	0.013
Ceramide (d18:1/18:2 OH)	1.65	1.45 - 1.88	0.015
Ceramide(d18:1/16:0)	1.57	1.36 - 1.77	0.018
L-Glutamic acid	1.54	1.38 -1.84	0.017
11-HETE	1.53	1.33 - 1.68	0.023
S-Adenosylhomocysteine	1.53	1.35 - 1.76	0.026
Dopamine	1.46	1.28 - 1.75	0.028
Niacinamide	1.43	1.22 -1.74	0.031
Indoxyl sulfate	1.42	1.09 - 1.64	0.034
Oleic acid	1.42	1.22 -1.73	0.033
Adipoylcarnitine	1.41	1.22 - 1.62	0.038
Carnosine	1.39	1.21 - 1.55	0.041
2-Octenoylcarnitine	1.38	1.18 - 1.53	0.042
Glycerol	1.37	1.14 - 1.49	0.044
9-Hexadecenoylcarnitine	1.34	1.11 -1.43	0.042
Inosine	1.33	1.08 - 1.42	0.032
Ceramide(d18:1/12:0)	1.22	1.06 -1.37	0.044
Phenylacetylglutamine	1.13	1.01 - 1.23	0.046
N-oleoylethanolamine	1.12	0.94 - 1.25	0.083
5-HETE	1.08	0.89 - 1.17	0.087
Ceramide(d18:1/16:1 OH)	1.06	0.92 -1.13	0.93
Homoarginine	1.03	0.93 - 1.21	0.086
DHC(18:1/16:0)	0.98	0.88 - 1.13	0.14
Homocitrulline	0.98	0.91 - 1.18	0.11
Octadecanoylcarnitine	0.95	0.86 - 1.15	0.21
Desmosterol	0.93	0.84 - 1.06	0.19
BMP (18:1/18:2)	0.93	0.89 - 1.11	0.32
BMP (18:1/16:1)	0.89	0.79 - 1.02	0.28
7-Dehydrocholesterol	0.87	0.75 - 0.96	0.042
4-Hydroxyphenyllactic acid	0.84	0.76 - 0.92	0.045
Tiglylglycine	0.83	0.71 - 0.91	0.048
Uridine	0.82	0.75 - 0.91	0.041
THC 18:1/24:1	0.78	0.59 - 0.88	0.039
Itaconic acid	0.75	0.73 - 0.86	0.046
2-Isopropylmalic acid	0.75	0.64 - 0.86	0.043
THC 18:1/22:0	0.73	0.58 - 0.91	0.039
2-Hydroxy-3-methylbutyric acid	0.73	0.64 - 0.79	0.032
THC 18:1/16:0	0.72	0.65 - 0.81	0.028
cis-aconitic acid	0.71	0.56 - 0.88	0.021

Butyrylcarnitine	0.71	0.59 - 0.83	0.025
PI(36:1)	0.68	0.48 - 0.83	0.022
Sphingosine 1-phosphate	0.67	0.54 - 0.71	0.019
PE(34:1)	0.65	0.53 - 0.78	0.038
PI(36:0)	0.59	0.44 - 0.69	0.035
CL(18:2/18:2/18:2/20:4)	0.57	0.43 - 0.72	0.034
2-Ketohexanoic acid	0.56	0.43 - 0.68	0.033
L-Aspartic acid	0.56	0.38 - 0.77	0.031
SM(d18:1/18:1)	0.55	0.43 - 0.64	0.028
SM(d18:1/16:0)	0.54	0.42 - 0.65	0.026
2-Hydroxyglutarate	0.53	0.41- 0.73	0.025
Phenyllactic acid	0.43	0.32 - 0.59	0.023
SM(d18:1/16:2)	0.38	0.29 - 0.51	0.018
Hippuric acid	0.37	0.23 - 0.49	0.031
LysoPC(18:0)	0.34	0.23 - 0.48	0.028
4-Hydroxyphenylpyruvic acid	0.32	0.21 - 0.45	0.026
Adipic acid	0.28	0.16 - 0.41	0.025
Retinol	0.27	0.15 - 0.38	0.023
Quinolinic Acid	0.25	0.13 - 0.44	0.018
PI (36:2)	0.21	0.07 - 0.59	0.013
Azelaic acid	0.18	0.05 - 0.54	0.0092
Isovalerylglycine	0.11	0.03 - 0.35	0.0074

Notes: Only the shared discriminating metabolites (n = 62, VIP > 1.2) in both cohorts were analyzed. Hazard raitos are per 1 SD increment of log2-transformed values. Adjusted for baseline age, BMI, hypertension status, hsCRP and LDL.

Abbreviations: CI: confidence interval; DHC: Dihexosylceramide; BMP: Bis(monoacylglycero)phosphate; THC: Trihexosylceramide; PI: Phosphatidylinositol; PE: Phosphatidylethanolamine; CL: Cardiolipin; LysoPC: Lysophosphatidylcholine; SM: Sphingomyelin; SD: Standard deviation; BMI: Body mass index; hsCRP: High-sensitive C-reactive protein; LDL: Low density lipoprotein.

Table S8. List of top 60 metabolites ranked by their contributions to classification accuracy of angina recurrence in random forest algorism in the discovery cohort.

Metabolites	Mean Decrease Accuracy
LysoPC(18:0)	0.018109
L-Palmitoylcarnitine	0.017854
Niacinamide	0.015946
PI(36:2)	0.013415
Phenyllactic acid	0.011504
Myristoylcarnitine	0.0095189
12-HETE	0.0088608
Ethanolamine	0.007752
Ceramide(d18:1/18:2 OH)	0.0073243
5-HETE	0.006545
Carnosine	0.006531
Pseudouridine	0.0063822
Adipic acid	0.0063679
L-Glutamic acid	0.0059979
Isovalerylglycine	0.0059631
2-Hydroxyglutarate	0.0056688
Indoxyl sulfate	0.0055896
Uridine	0.0055788
SM(d18:1/16:2)	0.0050651
CL(18:2/18:2/18:2/20:4)	0.0049634
4-Hydroxyphenylpyruvic acid	0.0049357
Taurine	0.0045388
Octadecanoylcarnitine	0.0044997
4-Hydroxyphenyllactic acid	0.0041855
SM(d18:1/18:1)	0.0041541
Azelaic acid	0.0041049
cis-aconitic acid	0.0039701
Ceramide(d18:1/16:0)	0.0037205
7-Dehydrocholesterol	0.0036799
Dopamine	0.0035369
7-Methylguanosine	0.0035305
Suberic Acid	0.0034894
S-Adenosylhomocysteine	0.0034502
SM(d18:1/16:0)	0.0033219
Oleic acid	0.0031762
2-Hydroxy-3-methylbutyric acid	0.0030991
Quinolinic Acid	0.0029005
Homoarginine	0.0027979
Citramalic acid	0.0027294
SM(d18:1/26:0)	0.0027087
Itaconic acid	0.0026826

CL(18:2/18:2/18:1/18:1)	0.0026004
Deoxyadenosine monophosphate	0.0025104
Glycerol	0.0024542
Cytidine	0.0023444
SM(d18:1/16:0 OH)	0.0023125
AICAR	0.0022879
Gluconic acid	0.002212
SM(d18:1/16:2 OH)	0.0020508
Phenylacetylglutamine	0.0019812
5-Methylthioadenosine	0.0019487
11-HETE	0.0019409
4-Hydroxybenzoic acid	0.0018024
CL(18:2/18:2/18:2/16:1)	0.0014679
Testosterone	0.0014472
L-Valine	0.0014459
Hypoxanthine	0.001427
Inosine	0.0013121
Ceramide(d18:1/12:0)	0.0013083
Methylcysteine	0.0012344

Table S9. List of top 60 metabolites ranked by their contributions to classification accuracy of angina recurrence in random forest algorism in the additional discovery cohort.

Metabolites	Mean Decrease Accuracy
SM(d18:1/18:1)	0.0079072
S-Adenosylhomocysteine	0.0074712
SM(d18:1/16:2)	0.0070143
Adipic acid	0.0069797
Hippuric acid	0.006865
Oleic acid	0.0066829
Ketoleucine	0.006609
Ceramide(d18:1/18:2 OH)	0.0065143
PI(36:2)	0.0064553
12-HETE	0.0062045
CL(18:2/18:2/18:1/18:1)	0.0061837
PC(18:1/22:6)	0.0061054
SM(d18:1/16:2 OH)	0.005986
Tetranor-PGEM	0.0059833
Itaconic acid	0.0058653
11,12-DiHETrE	0.0058053
Cholesterol	0.0056484
Deoxyadenosine monophosphate	0.0055196
N-oleoylethanolamine	0.0055009
Pseudouridine	0.0054913
Phenyllactic acid	0.0053606
PE(34:1)	0.0053136
2-Octenoylcarnitine	0.0052879
SM(d18:1/26:2 OH)	0.0052022
Tiglylglycine	0.0048982
2-Hydroxy-3-methylbutyric acid	0.0048597
Octadecanoylcarnitine	0.0048153
L-Valine	0.0047217
Ceramide(d18:1/12:0)	0.004683
SM(d18:1/16:0)	0.0046772
SM(d18:1/16:1)	0.0046733
2-Hydroxyglutarate	0.0046276
Sphingosine 1-phosphate	0.0046172
SM(d18:1/18:2)	0.0046158
Homocitrulline	0.0045916
Niacinamide	0.0045907
Betaine	0.0045301
PC(18:0/18:2)	0.004515
Ethanolamine	0.0044815
CL(18:2/18:2/18:2/22:6)	0.0041583
L-Tyrosine	0.0041413

Uridine	0.0040895
L-Palmitoylcarnitine	0.003996
7-Dehydrocholesterol	0.0039717
MHC(18:2/16:0)	0.0039567
PI(36:0)	0.0039031
LysoPC(18:0)	0.0039018
THC 18:1/24:1	0.0039
Carnosine	0.003893
Creatine	0.0038662
Quinolinic Acid	0.0038614
Adipoylcarnitine	0.0038482
SM(d18:1/16:1 OH)	0.0038408
Pyruvic acid	0.0038364
Arginine	0.0038293
7-Methylguanosine	0.0038277
4-Hydroxybenzoic acid	0.0037813
L-Glutamic acid	0.0036643
11-HETE	0.0036605
N-acetylserine	0.003194

Table S10. The MRM transitions, compound-dependent parameters, and their internal standards for the analysis of the metabolic predictors using stable isotope-dilution LC-MS/MS.

Metabolites	Polarity	Q1−Q3 (Quantifier)	Internal standards (IS)	Q1−Q3 (IS)	Q1−Q3 (Qualifier)	DP	EP	CE	CXP	RT (min)
L-Palmitoylcarnitine	Positive	400−187	L-Palmitoylcarnitine (N-methyl-D3)	403−187	400-85	70	10	32	15	3.83
12-HETE	Negative	319−179	12-HETE-d8	327−184	319−257	-100	-10	-30	-12	4.34
LysoPC(18:0)	Positive	524−184	PC(16:0/16:0)-d62	797−184	524−104	80	10	28	18	6.32
PI(36:2)	Negative	862−281	PI(17:0/20:4)	889−405	862−261	-56	-10	-70	-10	9.88
SM(d18:1/16:2)	Positive	701.6−79	SM(d18:1/18:1-d9)	738.6−189	701.6−184	120	10	85	10	11.12
Ceramide(d18:1/18:2 OH)	Positive	574.5−264	Ceramide(d18:1/18:1)-$^{13}C_{18}$	583−300	539−282	100	10	35	10	14.97

Notes: DP: Declustering potential; EP: Entrance potential; CE: Collision energy; CXP: Collision Cell Exit Potential; RT: Retention time; LysoPC: Lysophosphatidylcholine; PC: Phospholipid; PI: Phosphatidylinositol; SM: Sphingomyelin.

Table S11. The coefficients of 6 selected metabolic predictors in the multivariate logistic regression model of the external validation cohort.

Metabolic predictors	Coefficient	Standard error	*P* value
12-HETE	-2.269	0.784	<0.001
L-Palmitoylcarnitine	-1.536	0.603	0.004
LysoPC (18:0)	1.508	0.637	0.001
Ceramide (d18:1/18:2 OH)	-0.961	0.544	0.018
PI (36:2)	0.787	0.409	0.022
SM(d18:1/16:2)	0.836	0.402	0.037

A multi-biomarker model for predicting future angina recurrence was built using six selected metabolites by multivariate logistic regression in the external validation cohort (n = 130). The model was adjusted for baseline age, BMI, hsCRP and LDL.

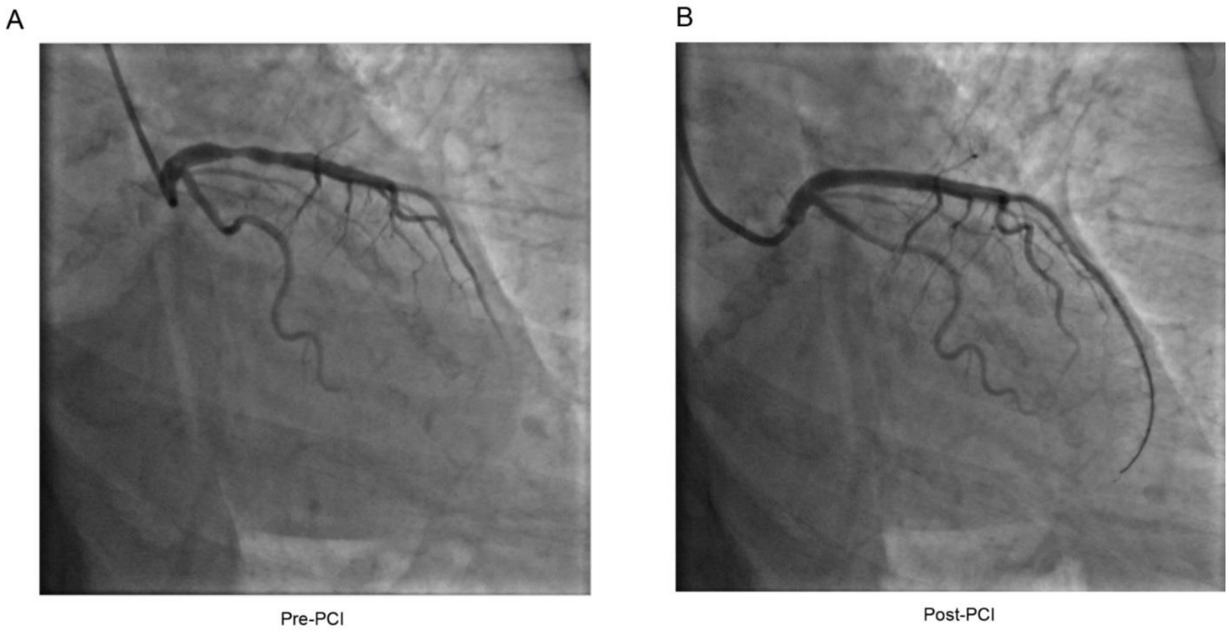

Figure S1. The representative images of the coronary angiography before and after the percutaneous coronary intervention (PCI) for a patient with stable angina. A. Pre-PCI image; B. Post-PCI image.

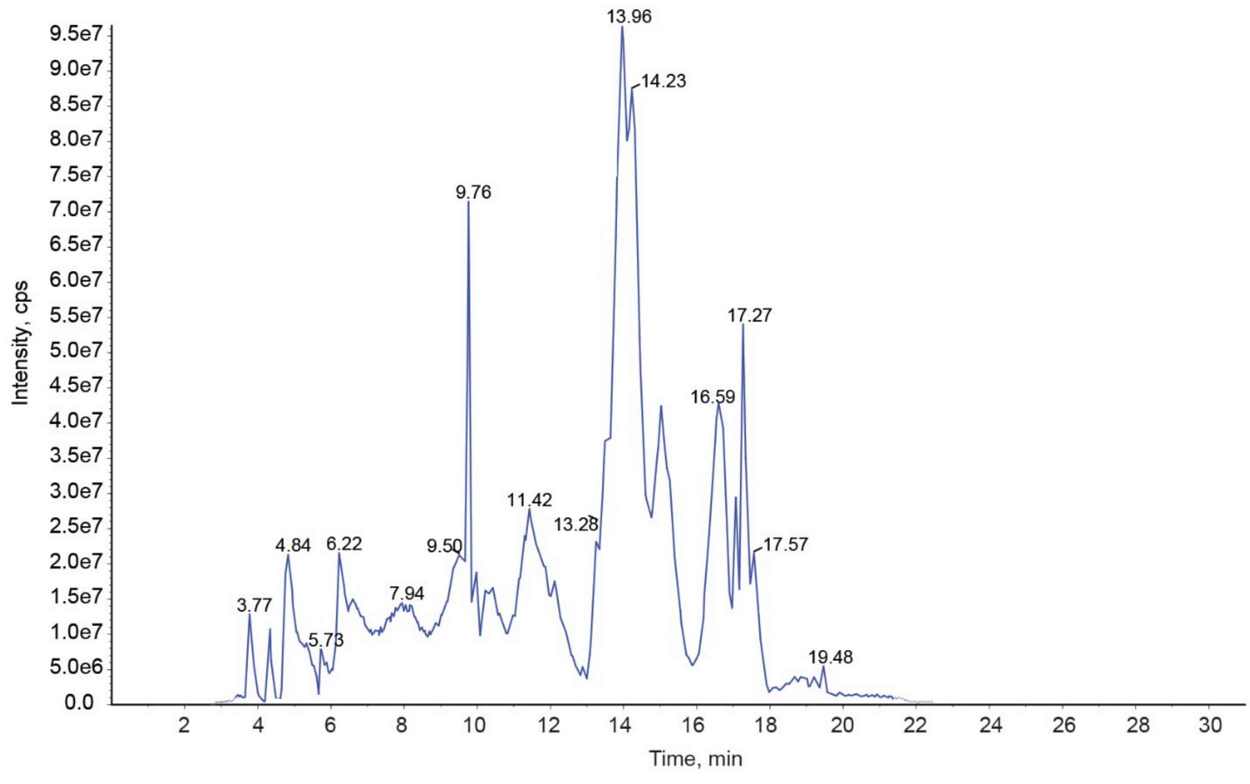

Figure S2. The representative chromatogram for the metabolomic analysis.

A total of 606 metabolites covering the main chemical classes in human plasma were targeted, and 407 metabolites were detected in all the participants without missing values.

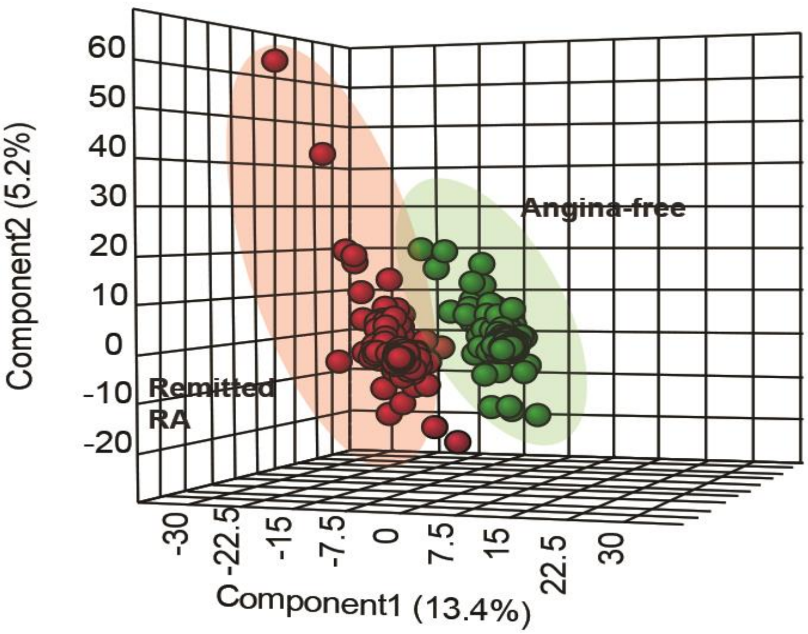

Figure S3. Partial least square discriminant analysis (PLS-DA) revealed the clear separation of the metabolic profiles in the plasma of remitted patients with angina recurrence within nine months after PCI from those of angina-free patients in the additional discovery cohort. N = 198 for patients with recurrent angina and N = 577 for angina-free.

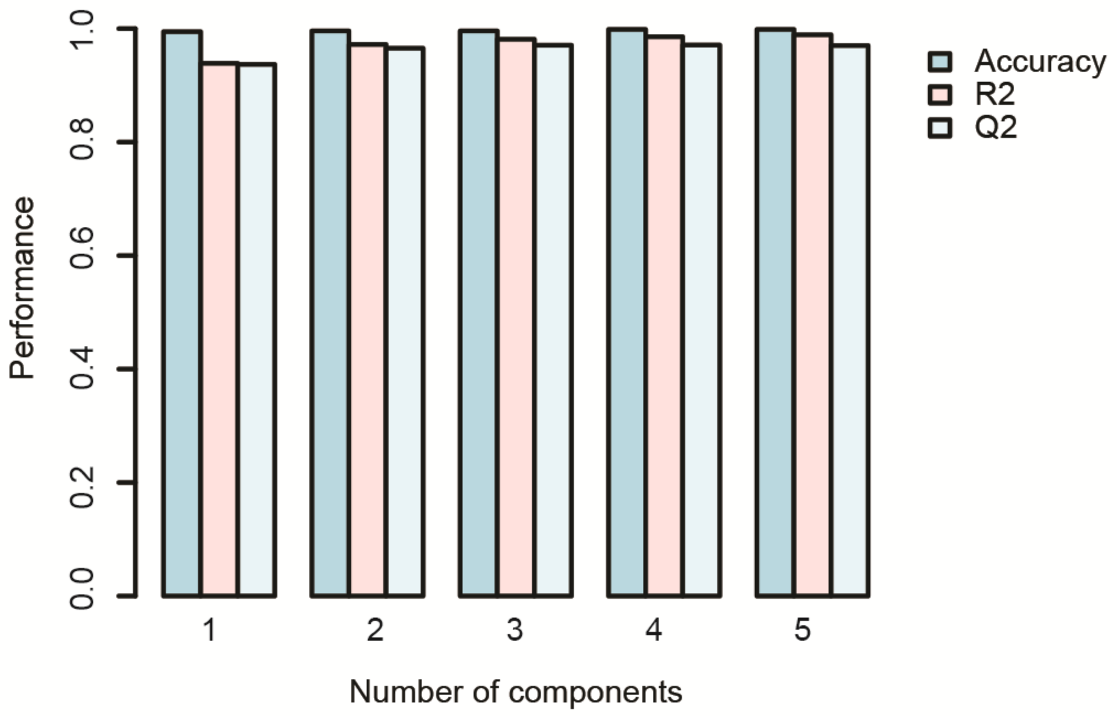

Figure S4. Assessment of partial least square discriminant analysis (PLS-DA) model for discriminating remitted patients with future angina recurrence from angina-free patients in the discovery cohort using leave-one-out cross-validation (LOOCV). Q^2 value > 0.4 in the first component (Component 1) was considered as a reliable model.

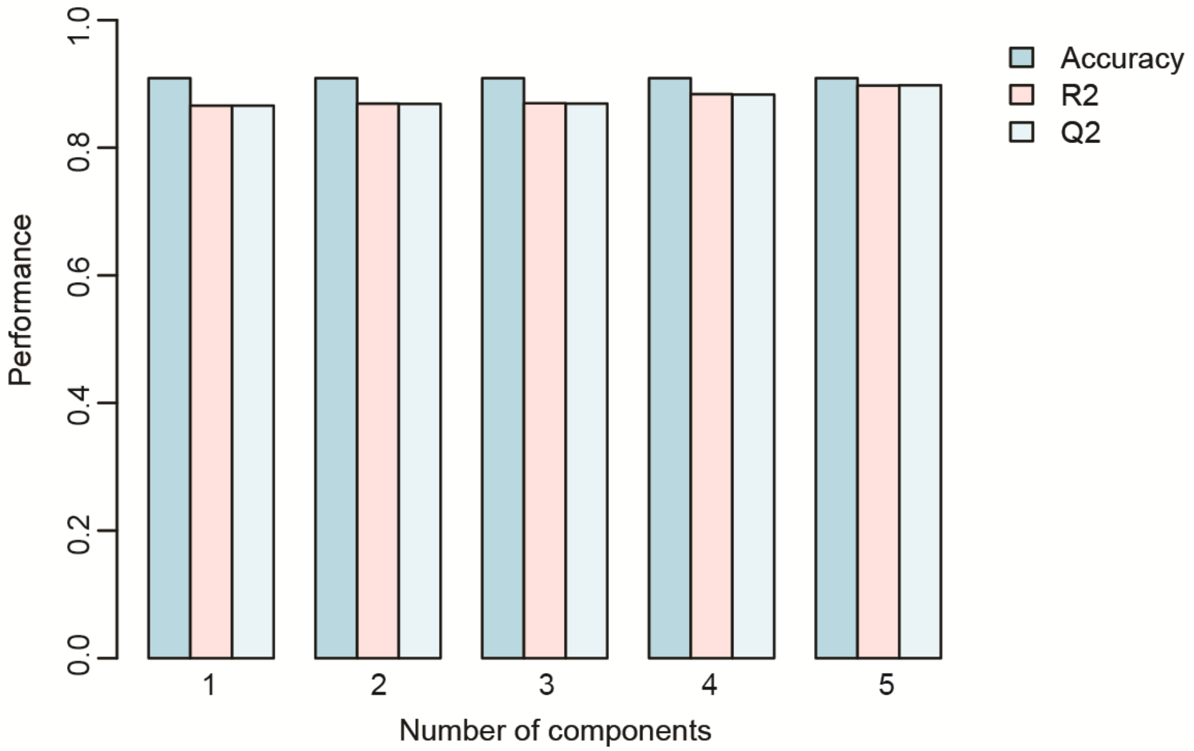

Figure S5. Assessment of the PLS-DA model for discriminating remitted patients with future angina recurrence from angina-free patients in the additional discovery cohort using leave-one-out cross-validation (LOOCV). Q^2 value > 0.4 in the first component was considered as a reliable model.

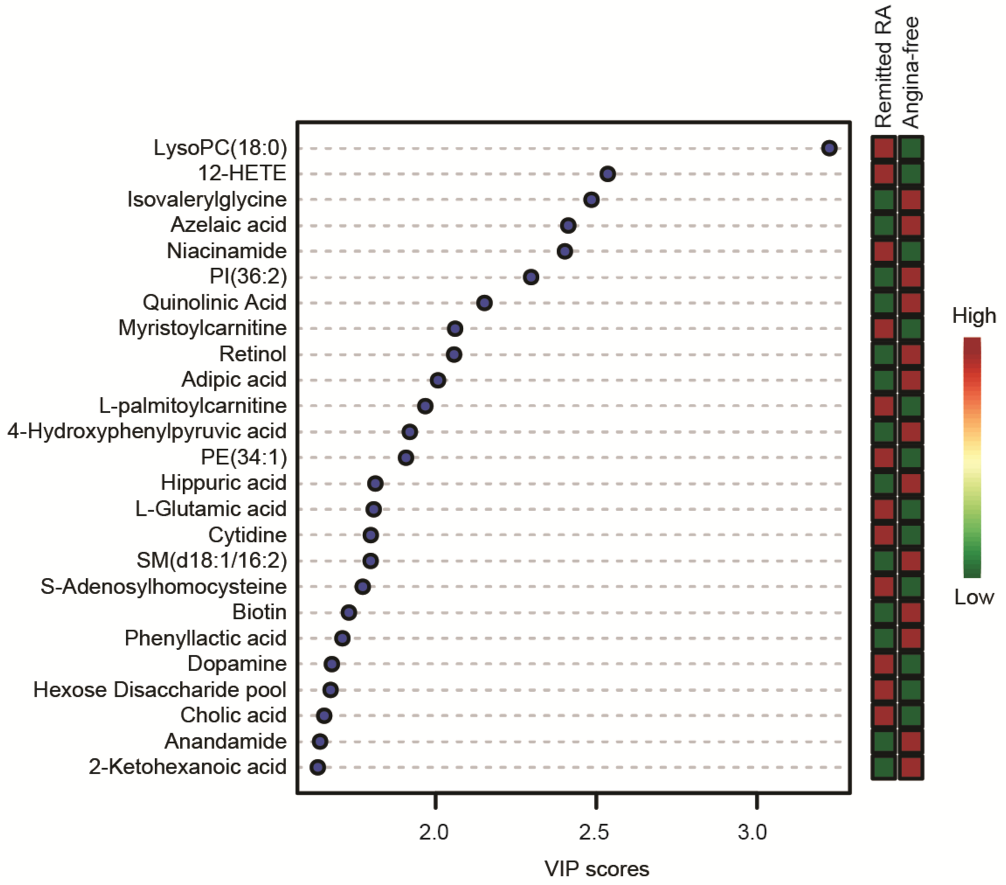

Figure S6. The top 25 discriminating metabolites in the plasma samples of remitted patients between recurrent angina and angina-free revealed by variable importance in projection (VIP) scores in the additional discovery cohort. N = 198 for remitted patients with recurrent angina (Remitted RA) and N = 577 for angina-free.

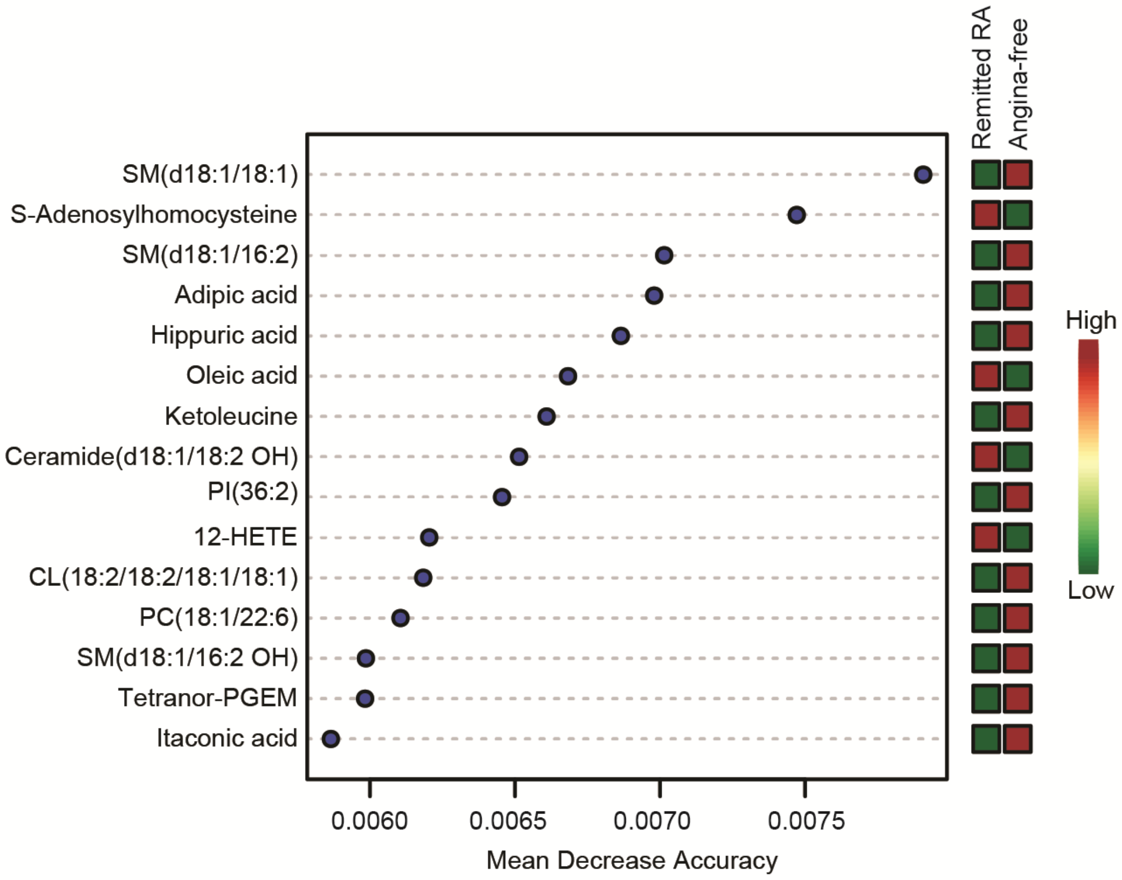

Figure S7. The top 15 metabolites ranked by their contributions to classification accuracy (Mean decrease accuracy, MDA scores) of angina recurrence in random forest algorism in the additional discovery cohort. n = 1000 trees. Notes: Remitted patients with recurrent angina: Remitted RA.

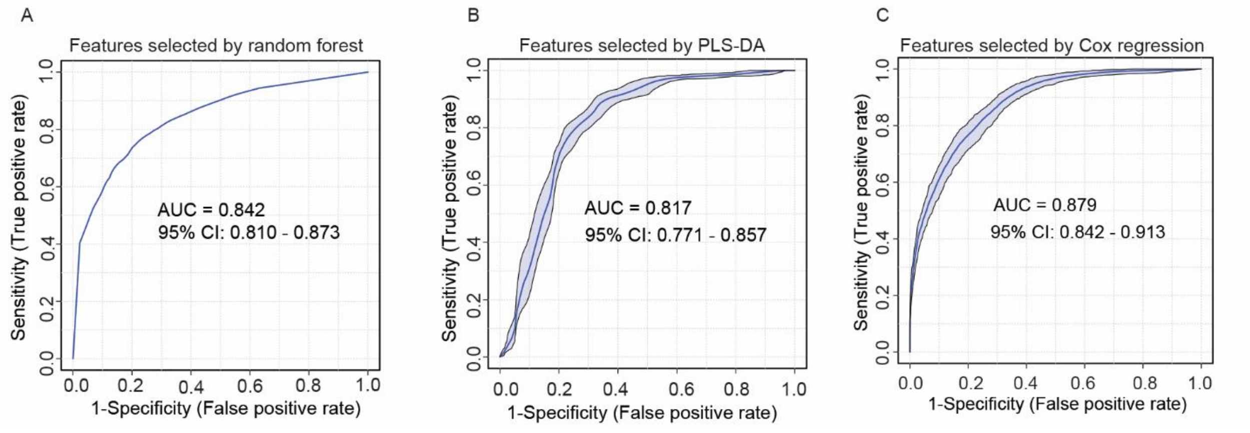

Figure S8. The prediction performance for future angina recurrence in the discovery cohort (n = 750) using the features selected by each individual machine learning algorithm. (A) Top 6 metabolites selected by random forest. (B) Top 6 metabolites revealed by PLS-DA. (C) Top 6 metabolites identified by Cox regression.

Top 6 metabolites in random forest were identified according to the mean decrease accuracy (MDA), which included SM(d18:1/18:1), S-Adenosylhomocysteine, SM(d18:1/16:2), adipic acid, hippuric acid, and oleic acid. Six metabolites in PLS-DA were selected according to the variable importance in projection (VIP) scores in the PLS-DA model (VIP scores), which included LysoPC(18:0), Phenyllactic acid, PI(36:2), L-Palmitoylcarnitine, Ethanolamine, and Urine. Top 6 metabolites selected by Cox regression analysis were 12-HETE, Isovalerylglycine, L-Palmitoylcarnitine, Azelaic acid, Myristoylcarnitine, and PI (36:2).

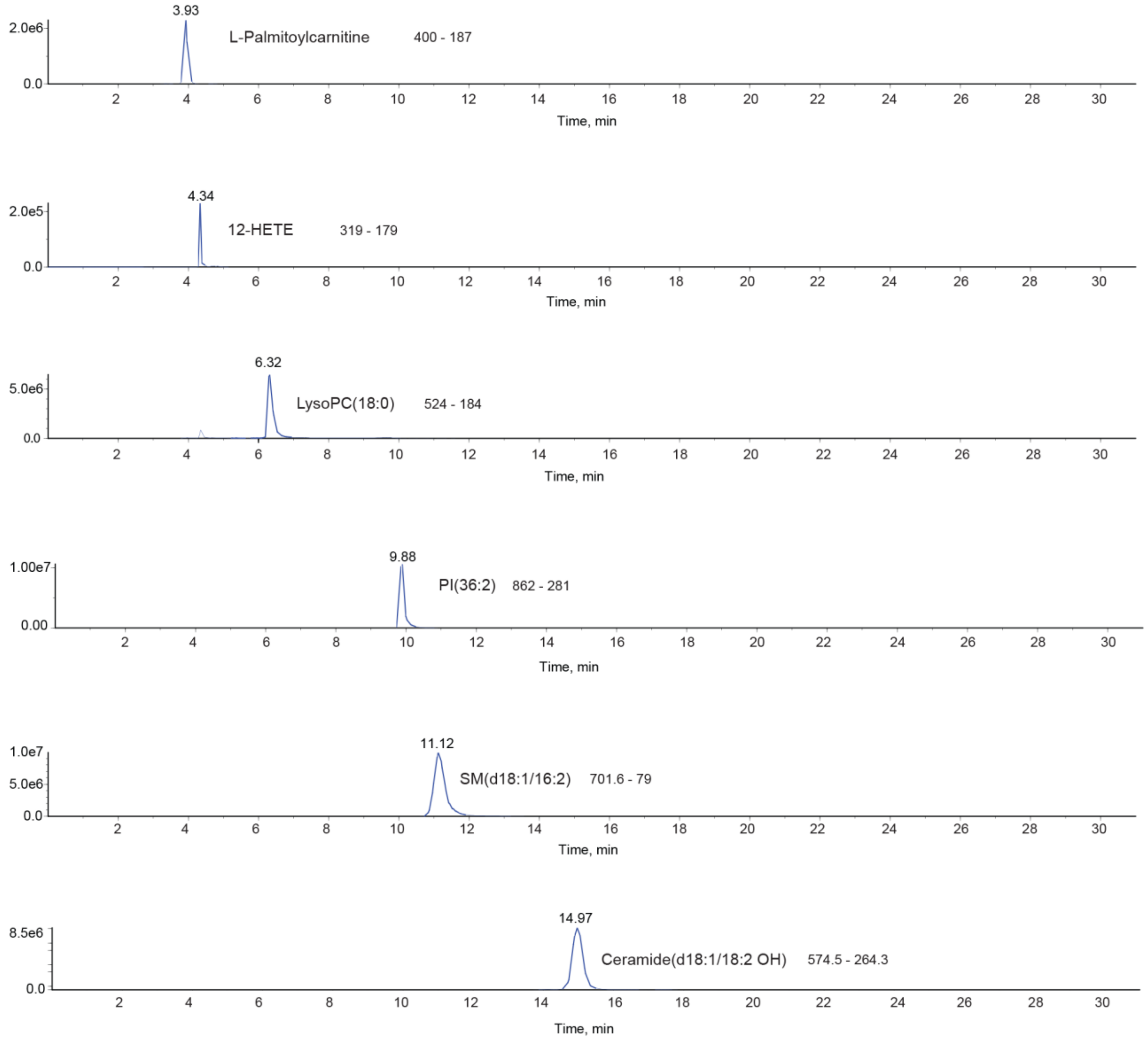

Figure S9. The representative LC-MS/MS chromatograms of metabolic predictors in a sample of the external validation cohort. The selected metabolites were quantified by stable isotope-dilution LC-MS/MS.

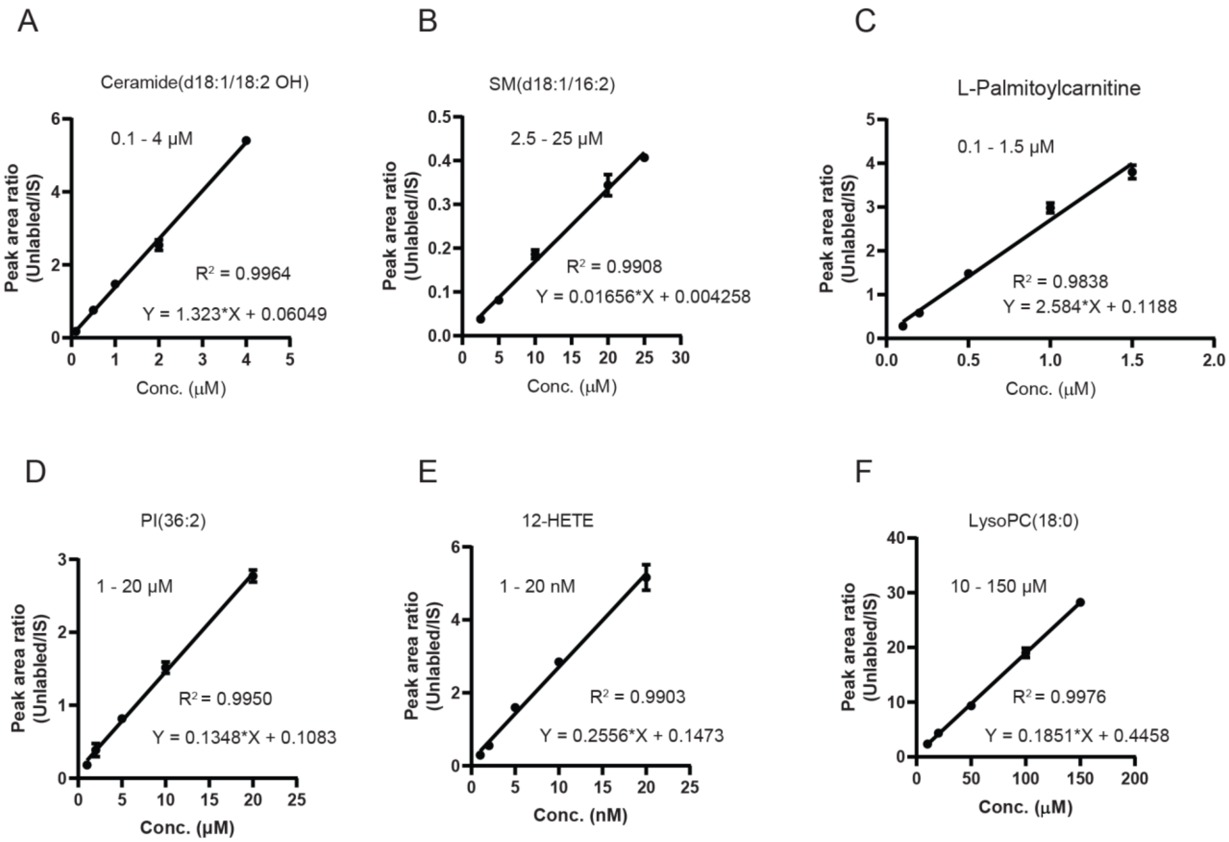

Figure S10. The standard curves and linear range for the selected metabolite predictors in the plasma matrix.
